# *Asianopis* gen. nov., a new genus of the spider family Deinopidae from Asia

**DOI:** 10.3897/zookeys.911.38761

**Published:** 2020-02-12

**Authors:** Yejie Lin, Lili Shao, Ambros Hänggi, John T.D. Caleb, Joseph K.H. Koh, Peter Jäger, Shuqiang Li

**Affiliations:** 1 Hebei Key Laboratory of Animal Diversity, College of Life Science, Langfang Normal University, Langfang 065000, China Langfang Normal University Langfang China; 2 Institute of Zoology, Chinese Academy of Sciences, Beijing 100101, China Institute of Zoology, Chinese Academy of Sciences Beijing China; 3 Naturhistorisches Museum Basel, Augustinergasse 2, CH 4001 Basel, Switzerland Naturhistorisches Museum Basel Basel Switzerland; 4 Zoological Survey of India, Prani Vigyan Bhawan, M-Block, New Alipore, Kolkata 700053, West Bengal, India Zoological Survey of India Kolkata India; 5 National Biodiversity Centre, National Parks Board, 259598, Singapore National Biodi­versity Centre, National Parks Board Singapore Singapore; 6 Senckenberg Research Institute, Senckenberganlage 25, 60325 Frankfurt a. M., Germany Senckenberg Research Institute Frankfurt a. M. Germany

**Keywords:** New combination, new species, species groups, systematics, taxonomy

## Abstract

A new genus of the spider family Deinopidae C.L. Koch, 1850 is described from Asia: *Asianopis* Lin & Li **gen. nov.**, with *A. zhuanghaoyuni* Lin & Li **sp. nov.** as the type species. The new genus is divided into two species groups, of which the *liukuensis*-group includes two species: *A. dumogae* (Merian, 1911) **sp. reval. comb. nov.** (♀) and *A. liukuensis* (Yin, Griswold & Yan, 2002) **comb. nov.** (♂♀); and the *zhuanghaoyuni*-group comprises five species: *A. celebensis* (Merian, 1911) **comb. nov.** (♂), *A. konplong* (Logunov, 2018) **comb. nov.** (♂), *A. wangi* Lin & Li **sp. nov.** (♂♀), *A. wuchaoi* Lin & Li **sp. nov.** (♂♀), and *A. zhuanghaoyuni* Lin & Li **sp. nov.** All previously described species are transferred from *Deinopis* MacLeay, 1839. *Deinopis scrubjunglei* Caleb & Mathai, 2014 is treated as a **junior synonym** of *Asianopis liukuensis***comb. nov.**

## Introduction

The spider family Deinopidae C.L. Koch, 1850 (Araneae, Deinopoidea), known as net-casting or ogre-faced spiders, is a small family that consisted of two genera and 64 species prior to the current study (World Spider Catalog 2019). The genus *Deinopis* was established by MacLeay (1839) based on *Deinopis lamia* MacLeay, 1839 (♂♀) from Cuba. The other genus, *Menneus*, was established by Simon (1876) based on *Menneus tetragnathoides* Simon, 1876 (♂) from Angola.

Ten species of Deinopidae were known from Asia: *Deinopis aruensis* Roewer, 1938 (♀) and *D. celebensis* Merian, 1911 from Indonesia; *D. fasciculigera* Simon, 1909 (♀) and *D. konplong* Logunov, 2018 (♂) from Vietnam; *D. scrubjunglei* Caleb & Mathai, 2014 (♂♀) from India; *D. gubatmakiling* Barrion-Dupo & Barrion, 2018 (juvenile), *D. labangan* Barrion-Dupo & Barrion, 2018 (♀), and *D. luzonensis* Barrion-Dupo & Barrion, 2018 (♀) from the Philippines; *D. kollari* Doleschall, 1859 (♂) from Myanmar and Malaysia; *D. liukuensis* Yin, Griswold & Yan, 2002 (♂♀) from China. Here, we describe a new genus and three new species, and present a molecular phylogenetic analysis of these spiders.

## Material and methods

All specimens were preserved in 80% ethanol. Metatarsi and tarsi were removed for preservation in 100% ethanol for subsequent molecular work. Epigynes were cleared in proteinase K at 56 °C to dissolve non-chitinous tissues for three hours. Specimens were examined under a LEICA M205C stereomicroscope. Photomicroscope images were taken with an Olympus C7070 zoom digital camera (7.1 megapixels). Laboratory habitus photographs were taken with a Canon 5D Mark III digital camera equipped with a Canon MP-E 65 mm lens. Photos were stacked with Helicon Focus (version 6.7.1) or Zerene Stacker (version 1.04) and processed in Adobe Photoshop CC 2018. Photographs of *Asianopis celebensis* comb. nov. were taken by a KEYENCE. Photographs of *Asianopis liukuensis* comb. nov. from India (i.e., the type materials of *D. scrubjunglei*) were taken using a Leica DFC500 HD camera mounted on a Leica M205A stereomicroscope.

All measurements are in millimetres. Eye sizes are measured as the maximum diameter from either the dorsal or frontal view. Leg measurements are given as follows: total length (femur, patella+tibia, metatarsus, tarsus). Copulatory duct turns are defined by the number of apparent loops on the lateral margin of the copulatory/fertilization duct complex in dorsal view. The length of the embolic tip fold is measured as from the beginning of the fold to the embolic tip (Fig. 22D, E). The terminology used in the text and figures follows Coddington et al. (2012). Distribution maps were generated using ArcMap software (version 10.2).

A total of 31 specimens of Deinopidae were collected for phylogenetic analysis (Suppl. material 1: Table S1). Sequences of seven specimens were from the National Center for Biotechnology Information (NCBI) public data, and the other 24 were from recent field collections. Whole genomic DNA was extracted from 2–4 legs using a TIANamp Genomic DNA kit (TIANGEN Inc., Beijing, China) following the manufacturer’s protocol. Seven gene fragments were amplified in 20-μL reactions: COI (~640 bp), 12S (~330 bp), 16S (~470 bp), 18S (~1700 bp), 28S (~1200 bp), H3 (~310 bp) and wnt (~330 bp). Primers and PCR conditions for each locus are listed in Suppl. material 1: Table S2. Sequence chromatograms were proofed and edited using Sequencher version 4.2 Demo (Gene Codes Corporation, Ann Arbor, MI USA). The COI, H3 and wnt fragments were translated in MEGA version 7 (Kumar et al. 2016) to check for the presence of stop codons. A representative of the family Uloboridae was used as the outgroup, with the corresponding sequences downloaded from NCBI. The complete list of 32 taxa and GenBank accession numbers are provided in Suppl. material 1: Table S1.

Multiple sequence alignments were carried out with MAFFT version 7.243 (Katoh and Standley 2013). Alignments of the protein-coding COI, H3 and wnt genes were produced using the L-INS-i method. As for the highly variable ribosomal genes, the E-INS-i method was used to generate alignments of 12S, 16S, 18S, and 28S. To exclude the ambiguously aligned regions, alignments of the ribosomal genes were processed with the program trimAl version 1.3 (Capella-Gutiérrez et al. 2009). The alignments are shown in the supplementary data.

The concatenated gene matrix was partitioned by gene using PartitionFinder version 1.1.1 (Lanfear et al. 2012). The best partitioning scheme was selected based on the Akaike information criterion (AIC) (Suppl. material 1: Table S3). Maximum likelihood (ML) analysis was performed using RAxML version 8.2.9 with a GTR + Γ + I model applied to each partition (Stamatakis 2014). One thousand non-parametric bootstrap replicates were conducted to obtain the best-scoring ML tree.

Bayesian analysis was performed using MrBayes version 3.2.6 (Ronquist et al. 2012). Two independent runs, each with four independent chains, were carried out for 20,000,000 generations and were sampled every 1,000 generations with a burn-in of 25%. Partitions and models followed the result of PartitionFinder. Convergence of the runs was determined with the standard deviation of split frequencies (<0.01). Effective sampling sizes (>200) of all parameters were checked in Tracer version 1.6 (Rambaut et al. 2014). A 50% majority-rule consensus tree was then constructed from the post-burnin sampled trees to estimate posterior probabilities (PP).

### Abbreviations

**ALE** anterior lateral eye

**AME** anterior median eye

**CD** copulatory duct

**CO** copulatory opening

**E** embolus

**EMA** embolic middle apophysis

**EO** embolic opening

**ETA** embolic terminal apophysis

**FD** fertilization duct

**MA** median apophysis

**MABL** median apophysis–basal lobe

**MADL** median apophysis–distal lobe

**MP** median plate

**PLE** posterior lateral eye

**PME** posterior median eye

**S** spermatheca

**SD** sperm duct

**SpD** spermathecal duct

**T** tegulum.

### Museum abbreviations

**HNU** Hunan Normal University, Changsha, China

**IZCAS** Institute of Zoology, Chinese Academy of Sciences, Beijing, China

**MMUE** Manchester Museum of the University of Manchester, UK

**NMB** Naturhistorisches Museum Basel, Basel, Switzerland

**SRC-ZSI** Southern Regional Centre, Zoological Survey of India, Kolkata, India

## Taxonomy

### Family Deinopidae C.L. Koch, 1850

#### Genus Asianopis

Taxon classificationAnimaliaAraneaeDeinopidae

Lin & Li gen. nov.

2DF06D83-9CC6-5EF2-90BE-9E565F1FE4B5

http://zoobank.org/C8CA3BB7-776C-4BB9-9E19-F819587E87AB

##### Type species.

*Asianopis zhuanghaoyuni* Lin & Li, sp. nov.

##### Etymology.

The generic name is a combination of the word “*Asia*”, referring to the distribution of the genus, and the generic name *Deinopis*. The gender is feminine.

##### Diagnosis.

*Asianopis* gen. nov. can be easily distinguished from *Deinopis* by the following characters: a prominent setal fringe can be found above the posterior median eyes in both sexes of *Asianopis* species (Fig. 4A, B), which is absent in *Deinopis* (Coddington et al. 2012: fig. 3a); the embolic tip of male *Asianopis* has an embolic middle apophysis (*liukuensis*-group, Fig. 21A), an embolic terminal apophysis or is weakly folded apically (*zhuanghaoyuni*-group, Fig. 21B–E), whereas none of these characters is present in *Deinopis* (Coddington et al. 2012: fig. 11m); the MADL in *Asianopis* is small and has a basal lobe, while in *Deinopis*, the median apophysis is larger than the MABL and covers the entire base (Coddington et al. 2012: fig. 11m); female chelicerae with many denticles between the promarginal and retromarginal teeth (Fig. 2F) or female chelicerae without denticles (Fig. 2H), in contrast, denticles are only at the center of any two adjoining retromarginal teeth in *Deinopis* (Coddington et al. 2012: fig. 5c); femora I enlarged proximally in *Asianopis* gen. nov. (*liukuensis* group, Fig. 2I) or not enlarged (*zhuanghaoyuni*-group, Fig. 2J), but they are enlarged distally in *Deinopis* (Coddington et al. 2012: fig. 3b); epigynal median plate lateral margins anchor-shaped in *Asianopis* gen. nov. (Figs 3A, 6A), but ellipsoid in *Deinopis* (Coddington et al. 2012: fig. 9b); SpD is consistently narrow in *Asianopis* gen. nov. (Figs 3B, 6B) but tapering in *Deinopis* (Coddington et al. 2012: fig. 9d).

**Figure 1.**
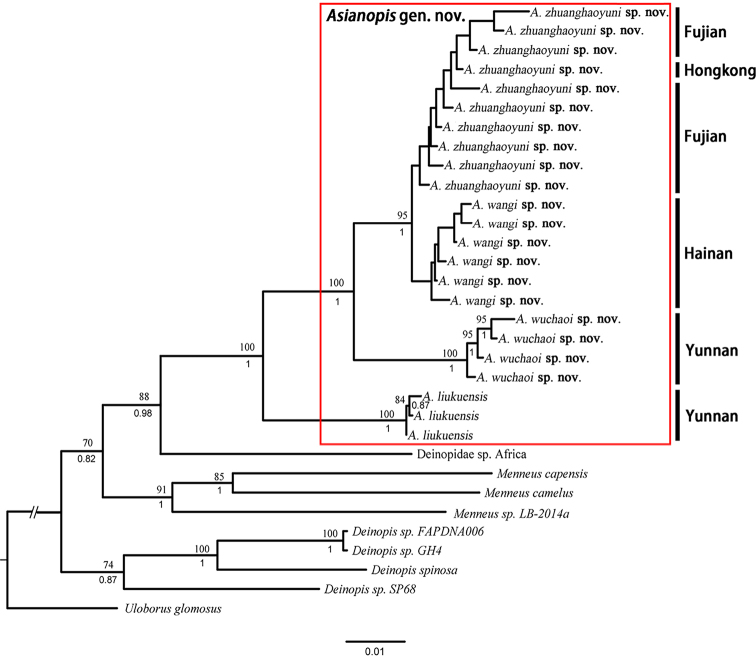
Phylogenetic tree of Deinopidae spiders based on 31 specimens. Numbers on nodes indicate Maximum Likelihood bootstrap values and Bayesian posterior probabilities.

**Figure 2.**
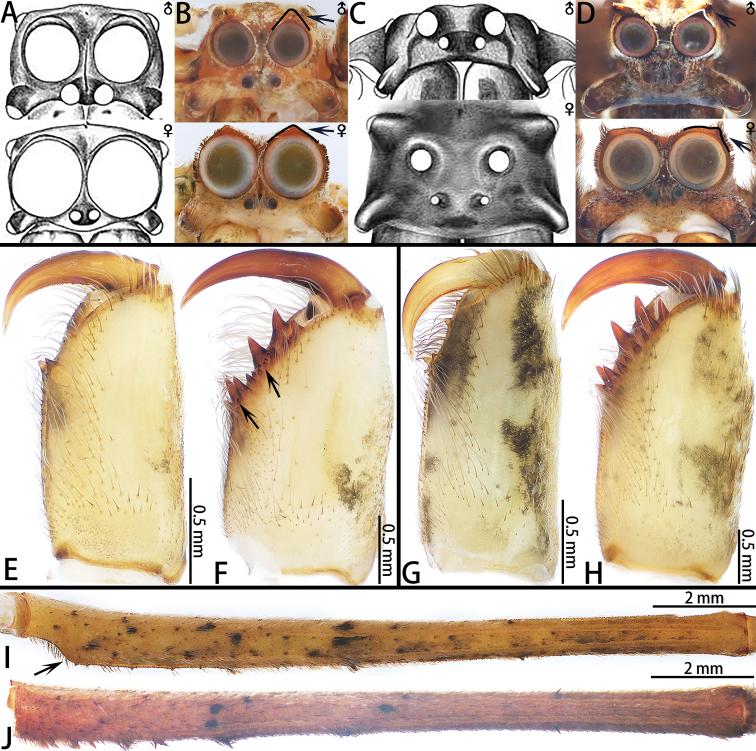
Prosoma (frontal view, upper ♂, lower ♀) (**A–D**), chelicerae (**E–H**) and leg I (**I–J**). Figures **A** and **C** modified from Coddington et al. (2012). **A***Deinopis spinosa***B***Asianopis liukuensis* comb. nov. **C***Menneus dromedarius***D***Asianopis zhuanghaoyuni* sp. nov. **E** Chelicerae of male *A. liukuensis* comb. nov. **F** Chelicerae of female *A. liukuensis* comb. nov. (Arrows indicate the denticles) **G** Chelicerae of male *A. zhuanghaoyuni* sp. nov. **H** Chelicerae of female *A. zhuanghaoyuni* sp. nov. **I** Left leg I of female *A. liukuensis* comb. nov. Arrow shows enlarged femur **J** Left leg I of female *A. zhuanghaoyuni* sp. nov.

##### Description.

**Male.** Total length 12.14–16.10 (*n* = 8), carapace pear-shaped, yellow-brown (*liukuensis*-group) or brown (*zhuanghaoyuni*-group) with white edge, white line extending from cephalic area to posterior margin and small spines sparsely distributed; fovea longitudinal, indistinct. Chelicerae with a promarginal tooth and one or two retromarginal teeth (*liukuensis*-group) or with four promarginal teeth and 2–6 retromarginal teeth (*zhuanghaoyuni*-group), no denticles. Endites and labium brown, distally white; sternum diamond-shaped, brown with median light band and few small spines. Legs brown, ventrally with black pattern and short spines, leg formula 1243. Opisthosoma cylindrical, brown or dark-brown with small black spots and irregular pattern. Cribellum entire, spinnerets brown (Figs 4, 10, 13, 16).

**Female.** Total length 14–24 (*n* = 13). Chelicerae with four promarginal teeth and seven retromarginal teeth, many denticles in between the promarginal and retromarginal teeth (*liukuensis*-group) or four promarginal teeth and 8–13 retromarginal teeth, without denticles (*zhuanghaoyuni*-group). Appearance of carapace, opisthosoma and legs as in male but femora of legs I enlarged basally (*liukuensis*-group) (Fig. 2I).

Male palpal tibia longer than cymbium; cymbium almost round; tegulum distinctly wider than the diameter of embolic coil (*liukuensis*-group) or tegulum obscured by embolic coil (*zhuanghaoyuni*-group) (Figs 17, 18); embolus long and strongly coiled around MA, embolic base beginning at 7–8 o’clock position, coiled 1200° (*liukuensis*-group) or more than 1500° (*zhuanghaoyuni*-group), embolic tip straight (*liukuensis*-group) or widened subapically, folded and without apophysis (*zhuanghaoyuni*-group); MA small, directed at 7–8 o’clock position, with two lobes, a small lobe at the base, and a narrow distal lobe with two apophyses (*liukuensis*-group) or large, with two lobes, a large lobe at the base and a kidney-shaped distal lobe (*zhuanghaoyuni*-group).

Epigyne with anchor-shaped median plate, CO distinct, CD with three turns, S oval, SpD consistently wide (*liukuensis*-group) or with a well-developed MP, obscuring CO, CD with 7–8 turns, S oval, SpD consistently thin (*zhuanghaoyuni*-group).

##### Molecular phylogeny.

The molecular phylogenetic analysis indicates with strong support that all the species in this study do not belong to *Deinopis*. Based on the 4893 bp-aligned sequences of seven gene fragments, the ML and Bayesian analyses produced the same topology, showing a split of a Southwest China clade from other clades and is strongly supported (Bootstrap value: 88; PP: 0.98) (Fig. 1). Our results are consistent with the results of Chamberland et al. (2018) who conducted a global phylogenetic analysis of *Deinopis*. Therefore, the Southwest China clade can be classified as a new genus with strong support (Bootstrap value: 100; PP: 1). Although intraspecific support values are low in both ML and Bayesian analyses results, basal nodes are strongly supported, including the sister relationship of *A. wangi* Lin & Li, sp. nov. & *A. zhuanghaoyuni* Lin & Li, sp. nov. (Bootstrap value: 95; PP: 1).

##### Natural habitat.

All the species of *Asianopis* gen. nov. were collected from bushes in low-elevation forests.

##### Composition.

This new genus comprises two species groups: the *liukuensis*-group with two species: *A. dumogae* (Merian, 1911) sp. reval. comb. nov. and *A. liukuensis* (Yin, Griswold & Yan, 2002) comb. nov. and the *zhuanghaoyuni*-group with five species: *A. celebensis* (Merian, 1911) comb. nov., *A. konplong* (Logunov, 2018) comb. nov., *A. wangi* sp. nov., *A. wuchaoi* sp. nov., and *A. zhuanghaoyuni* sp. nov.

##### Distribution.

China (Fujian, Yunnan, Hong Kong, Guangxi, Hainan), India, Indonesia, and Vietnam.

#### The *liukuensis*-group

##### Asianopis dumogae

Taxon classificationAnimaliaAraneaeDeinopidae

(Merian, 1911), sp. reval. comb. nov.

AB4BA7F0-0635-5CC3-BBA6-9EAC7114A56E

Fig. 3

Dinopis dumogae Merian, 1911: 171 (♀ only, ♂ mismatched).

###### Type material examined.

1♀ (NMB-ARAN-00514a), “Wald bei Duluduo”, Sulawesi Utara, forest near Duluduo, 00°31'33"N, 123°57'10"E, Sulawesi, Indonesia.

###### Diagnosis.

This species can be distinguished from *A. liukuensis* comb. nov. by the MP nearly covering the CO, S round, and the overall equal thickness of the CD (Figs 4, 6).

**Figure 3.**
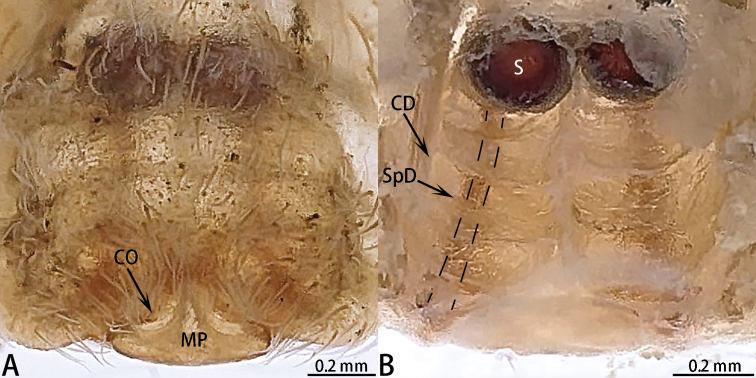
*Asianopis dumogae* sp. reval. comb. nov., female type. **A** Epigyne **B** Vulva, dorsal view.

**Figure 4.**
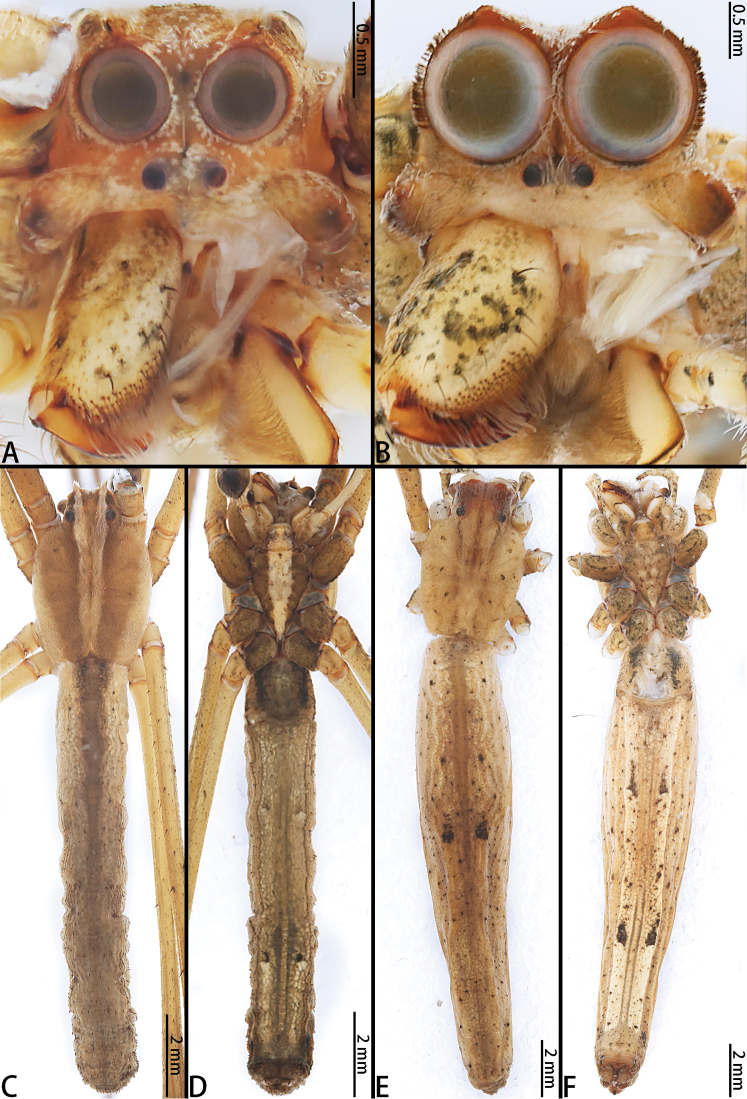
*Asianopis liukuensis* comb. nov., male from Xishuangbanna and female from Jianfengling. **A** Male prosoma, frontal view **B** Female prosoma, frontal view **C** Male habitus, dorsal view **D** Male habitus, ventral view **E** Female habitus, dorsal view **F** Female habitus, ventral view.

**Figure 5.**
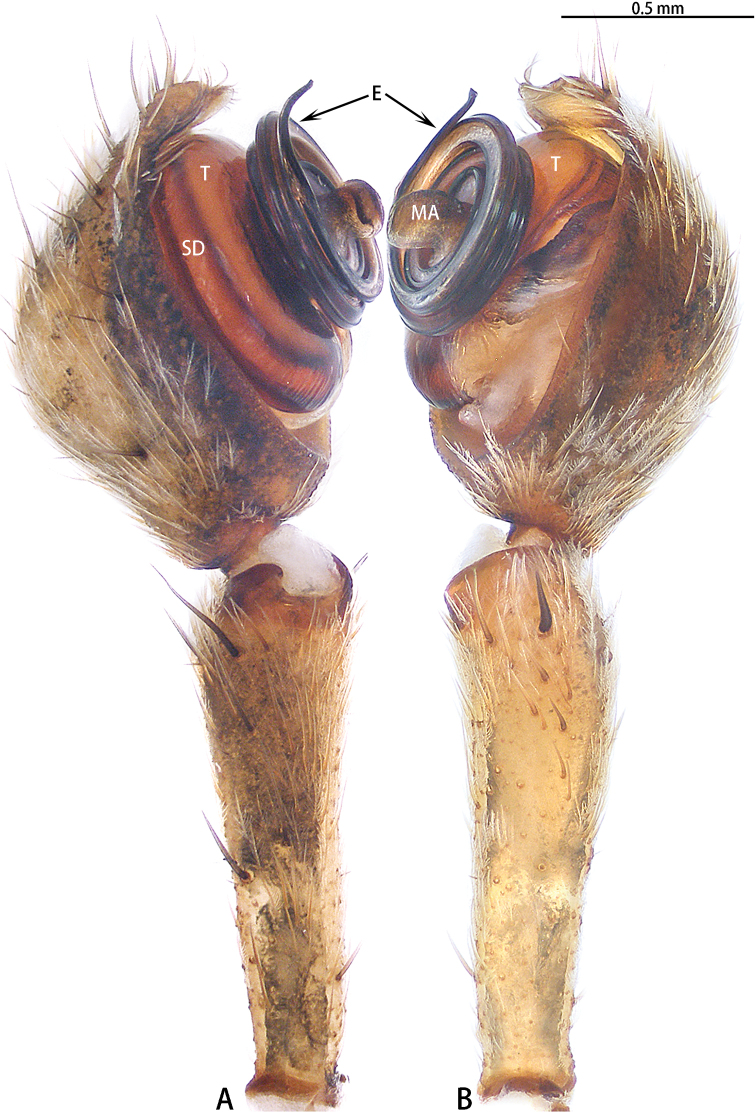
*Asianopis liukuensis* comb. nov., left palp, male from Xishuangbanna. **A** Prolateral view **B** Retrolateral view.

###### Description.

**Figure 6.**
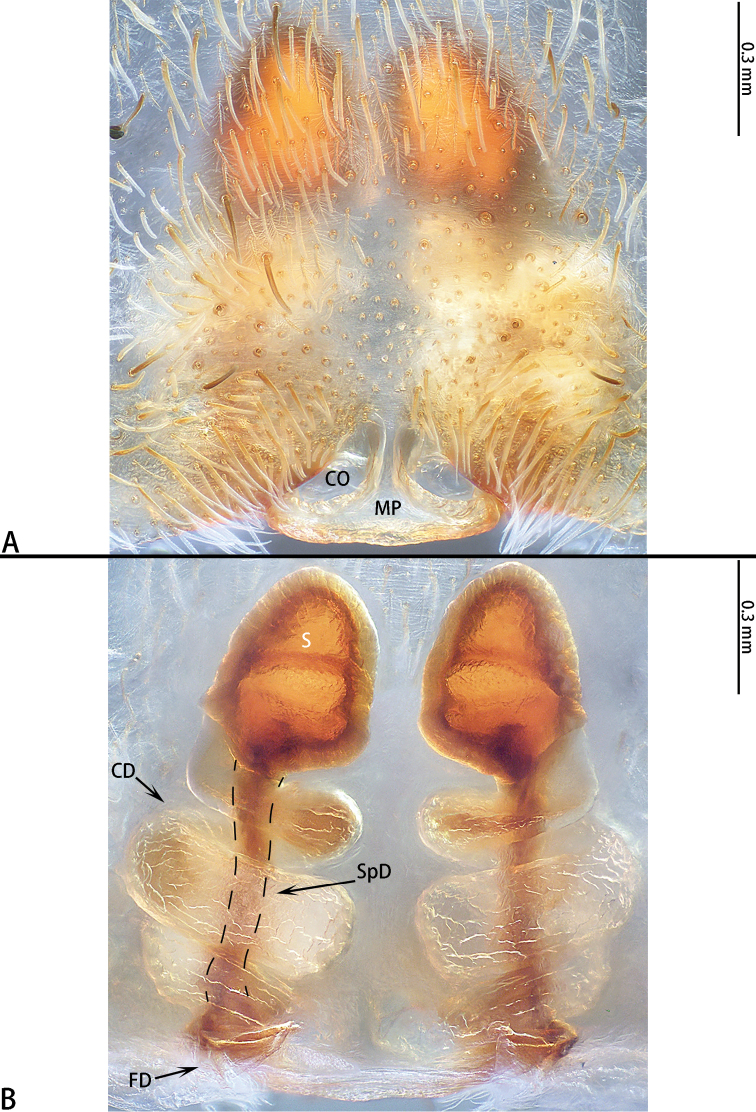
*Asianopis liukuensis* comb. nov., female from Jianfengling. **A** Epigyne **B** Vulva, dorsal view.

See Merian (1911). Photos of the epigyne of the syntype are given in Figure 6.

###### Distribution.

Indonesia (North Sulawesi).

###### Comments.

Merian (1911) reported *D. celebensis* based on three specimens from different localities in Sulawesi, Indonesia. One male (NMB-ARAN-00514b, “Zentral-Celebes, nördlich vom Golf von Bone”, South Sulawesi, north of the Gulf of Boni (precise locality not known), one female from North Sulawesi (NMB-ARAN-00514a, “Wald bei Duluduo”, Sulawesi Utara, forest near Duluduo, 00°31'33"N, 123°57'10"E and one female from Central Sulawesi (NMB-ARAN-00514c, Larga, südlich vom Posso-See, unterhalb Patiro Rano, bei 900 m, Central Sulawesi, south of Lake Poso at an elevation of 900 m (the localities “Larga” and “Patiro Rano” could not be located on maps; the epigyne of this specimen is missing, but the specimen is clearly larger than the others).

Merian (1911) stated that the male and the females may not represent the same species and suggested the name *D. celebensis* for the male, and *D. dumogae* for the female. According to the International Code of Zoological Nomenclature (International Commission on Zoological Nomenclature 1999: Article 11.5.1), such conditionally proposed species names are potentially available as valid names if published before 1961. The species has not been listed in any of the catalogues. We examined the types and concluded the male and the two females are indeed three different species. The palp of the male *D. celebensis* exhibits features of the *zhuanghaoyuni* group: the tegulum is obscured by the embolic coil, and the embolus is long and strongly coiled around the MA. The female from North Sulawesi (Doloduo) has features of the *liukuensis* group: an anchor-shaped median plate, CO distinct, CD with three turns. Thus, we revalidated the female *D. dumogae* as *Asianopis dumogae* (Merian, 1911), sp. reval. comb. nov.

##### Asianopis liukuensis

Taxon classificationAnimaliaAraneaeDeinopidae

(Yin, Griswold & Yan, 2002) comb. nov.

0B61E438-BC43-5540-8A8D-04906837BEB3

Figs 2B, E, F, I , 4 , 5 , 6 , 7 , 8 , 19 , 21A , 22A, G , 23

Deinopis liukuensis Yin et al., 2002: 610, figs 1–7 (♂♀)
Deinopis liukuensis Zhang & Wang, 2017: 238 (♂♀)
Deinopis scrubjunglei Caleb & Mathai, 2014: 2, figs 1–20 (♂♀) syn. nov.

###### Type.

***Holotype*.** ♂ (HNU, no. 00-LK-1, lost), China, Yunnan Province, Liuku, Mt Gaoligong, 25°30'48"N, 98°30'36"E, elevation ca 800 m, 26.VI.2000, Heng-Mei Yan leg.

###### Type materials of *Deinopis scrubjunglei* examined.

♂ (SRC-ZSI I/SP 19), Madras Christian College, Chennai, Tamil Nadu, 12°55'12.7"N, 80°07'24.6"E, elevation ca 32 m, 5.XII.2013, John Caleb T.D. leg.; ♀ (SRC-ZSI I/SP 20), 22.IV.2014, same location, John Caleb T.D and Karthy leg.

###### Other material examined.

2♂, China, Yunnan Province, Xishuangbanna Dai Autonomous Region, rubber tree plantation near Jinghong City, 28.IV.2016, Chaotai Wei leg.; 1♀, China, Hainan Island, Ledong County, Jianfengling National Park, 13.VII.2019, Zixuan Lin leg.

###### Diagnosis.

This species can be distinguished from other congeners by the distinct female copulatory opening, oval S, and CD tapering from the copulatory opening to spermatheca (Figs 6, 8).

###### Description.

See Yin et al. (2002) and Caleb and Mathai (2014).

###### Distribution.

China (Yunnan, Guangxi, Hainan), India.

###### Comments.

**Figure 7.**
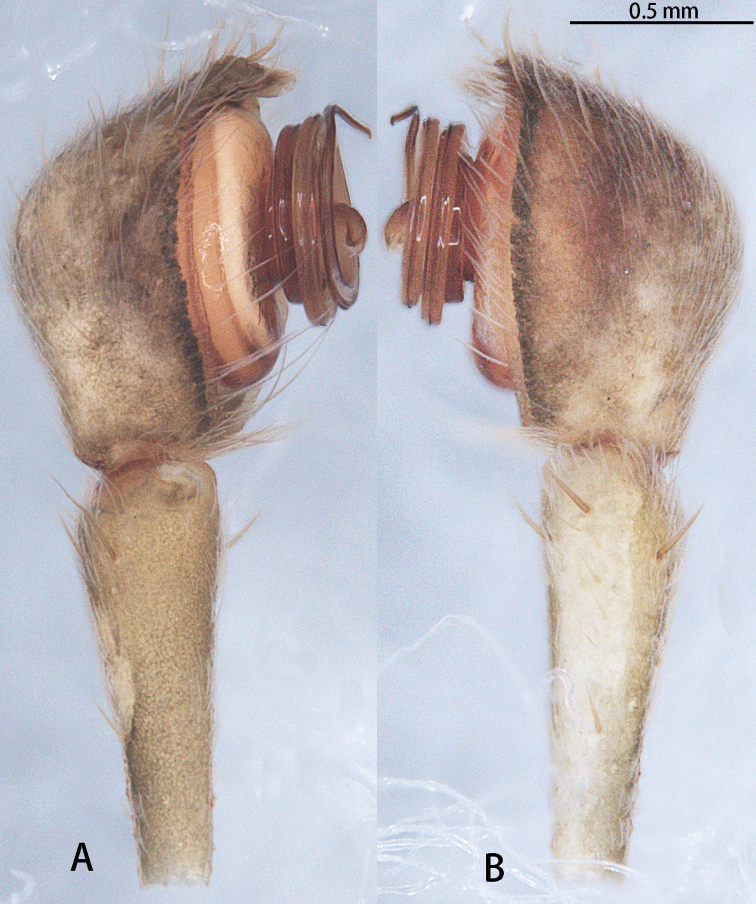
*Asianopis liukuensis* comb. nov., left palp, holotype male of *Deinopis scrubjunglei* syn. nov. **A** Prolateral view **B** Retrolateral view.

**Figure 8.**
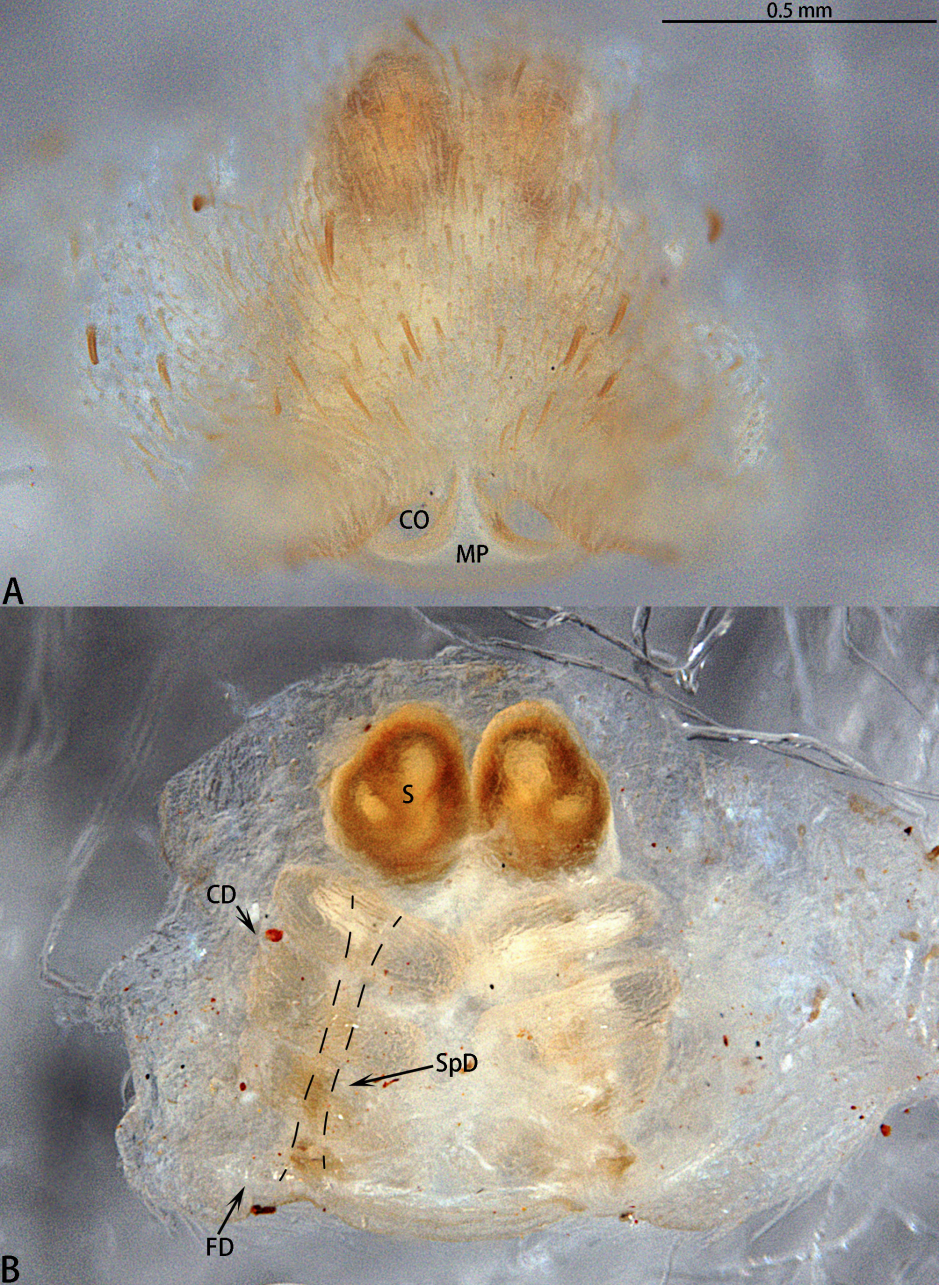
*Asianopis liukuensis* comb. nov., paratype female of *Deinopis scrubjunglei* syn. nov. **A** Epigyne **B** Vulva, dorsal view.

Type materials of *D. scrubjunglei* syn. nov. were examined and no differences between *A. liukuensis* and *D. scrubjunglei* were observed. Thus, we consider *D. scrubjunglei* to be a synonym of *A. liukuensis*, and the figures of *D. scrubjunglei* are given for comparison (Figs 7, 8, 19C).

#### The *zhuanghaoyuni*-group

##### Asianopis celebensis

Taxon classificationAnimaliaAraneaeDeinopidae

(Merian, 1911) comb. nov.

08ADBAE9-C3A2-5D4F-A2FF-133634EEA3BD

Fig. 9A–F

Dinopis celebensis Merian, 1911: 167, figs A, B (♂only, ♀ mismatched).

###### Type material examined.

♂ (NMB), NMB-ARAN-00514b, “Zentral-Celebes, nördlich vom Golf von Bone”, South Sulawesi, north of the Gulf of Boni (precise locality not known).

###### Diagnosis.

**Figure 9.**
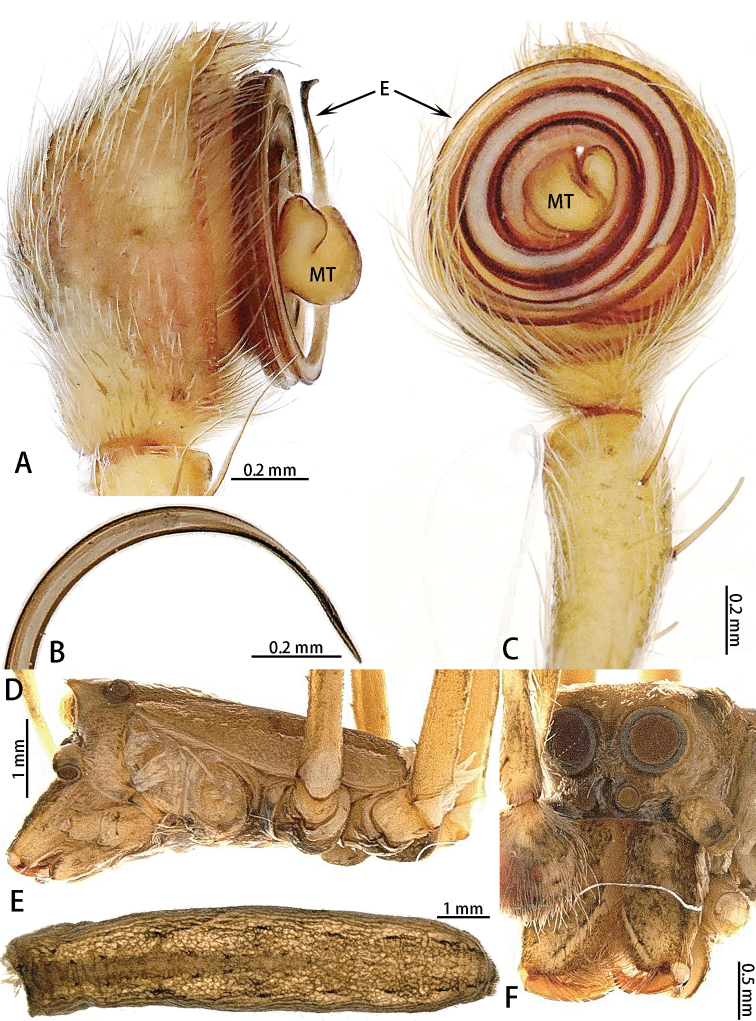
*Asianopis celebensis* comb. nov., male type. **A** Male right palp, prolateral view **B** Embolic tip **C** male left palp (embolic tip detached), retrolateral view **D** Male prosoma, lateral view **E** Male opisthosoma, dorsal view **F** Male prosoma, frontal view.

The male can be distinguished from other congeners by having the distal lobe of the MA distinctly smaller than the basal lobe; in other *Asianopis* spp., the distal lobe is slightly smaller than the basal lobe (Fig. 9A, C).

###### Description.

See Merian (1911). Photos of holotype male habitus and palps are shown in Fig. 9A–F.

###### Distribution.

Indonesia (Sulawesi).

###### Comments.

One male and two females were types for *Asianopis celebensis* (Merian, 1911) comb. nov. after Merian (1911). Based on the current study, one type female from North Sulawesi is *Asianopis dumogae* (Merian, 1911) sp. reval. comb. nov., and the other type female from South Sulawesi is a member of the *zhuanghaoyuni*-group, but its status at the species level is uncertain because of the missing epigyne.

##### Asianopis konplong

Taxon classificationAnimaliaAraneaeDeinopidae

(Logunov, 2018) comb. nov.

884BE3FF-059D-511D-BD11-8D1BBC06B116

Deinopis konplong Logunov, 2018: 141, figs 1–7 (♂).

###### Type.

Holotype ♂ (MMUE, G7579.37) from Vietnam, Kon Tum Province, Kon Plong District, 14 km north of Kon Plong, 14°43'20"N, 108°18'59"E, elevation ca 1030 m, 3–12.VI.2016, A.A. Abramov leg. Not examined.

###### Diagnosis.

This species can be distinguished from other *Asianopis* species by the short palp (ratio of the length of the palpal tarsus to the length of the cymbium: 1:1) and upturned embolic tip (Logunov 2018: fig. 4).

###### Description.

See Logunov (2018).

###### Distribution.

Vietnam (Kon Tum).

##### Asianopis wangi

Taxon classificationAnimaliaAraneaeDeinopidae

Lin & Li sp. nov.

CCC7E087-4B38-54C6-BFC6-F70A71B74D98

http://zoobank.org/64A4C3D1-03A5-4D7A-B2E6-E30EA28DC41C

**Figure 10.**
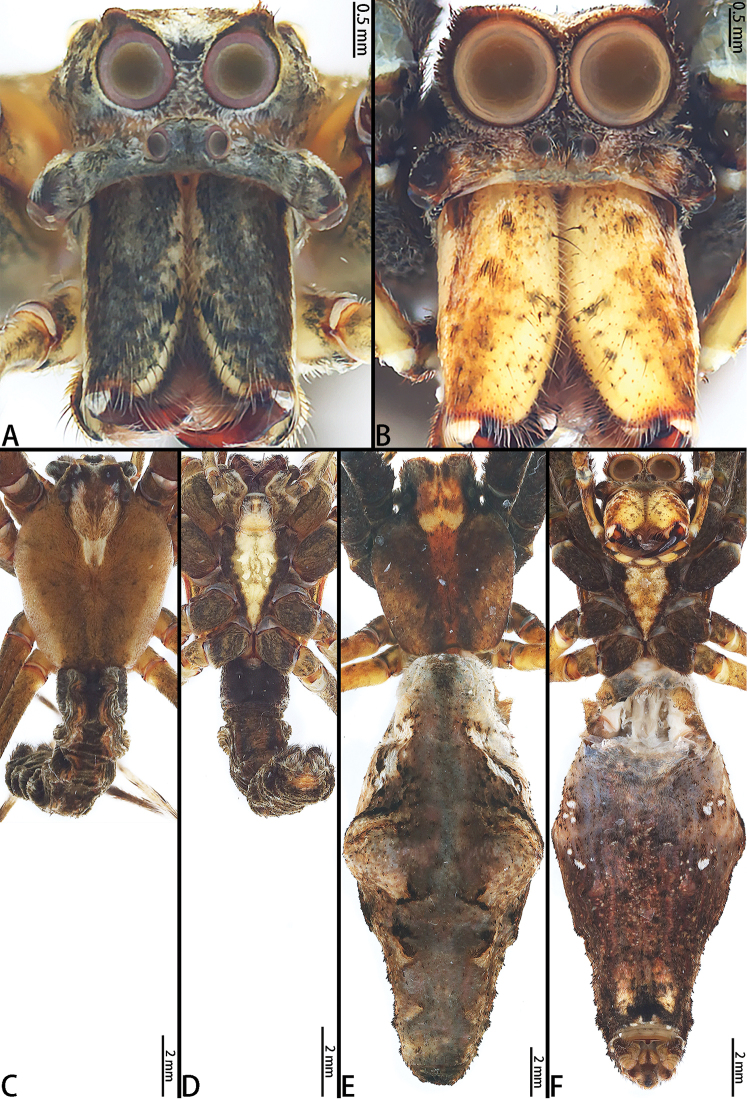
*Asianopis wangi* sp. nov., male holotype and female paratype. **A** Male prosoma, frontal view **B** Female prosoma, frontal view **C** Male habitus, dorsal view **D** Male habitus, ventral view **E** Female habitus, dorsal view **F** Female habitus, ventral view.

**Figure 11.**
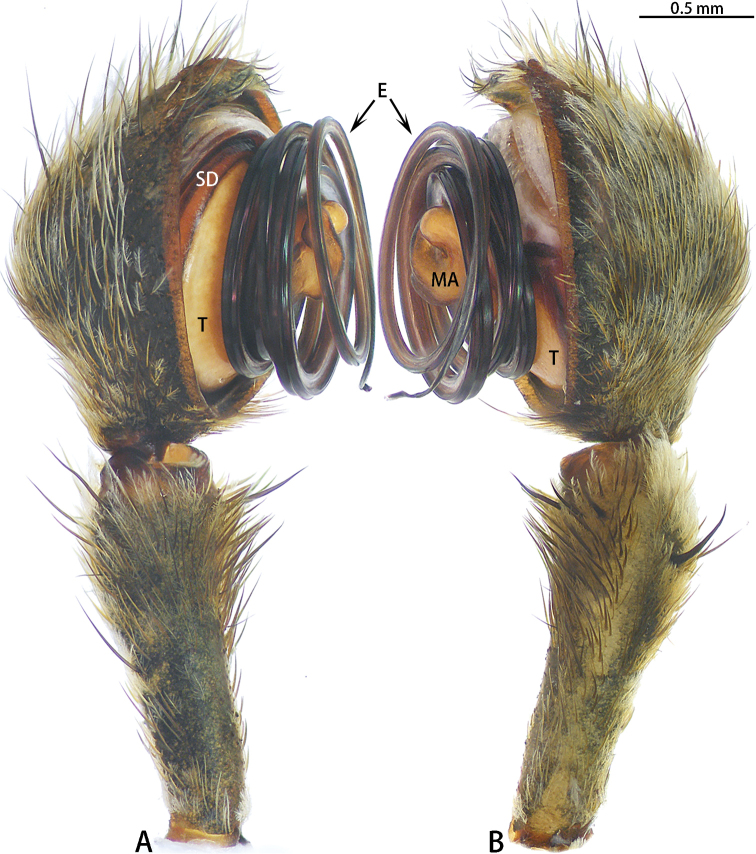
*Asianopis wangi* sp. nov., left palp, male holotype. **A** Prolateral view **B** Retrolateral view.

**Figure 12.**
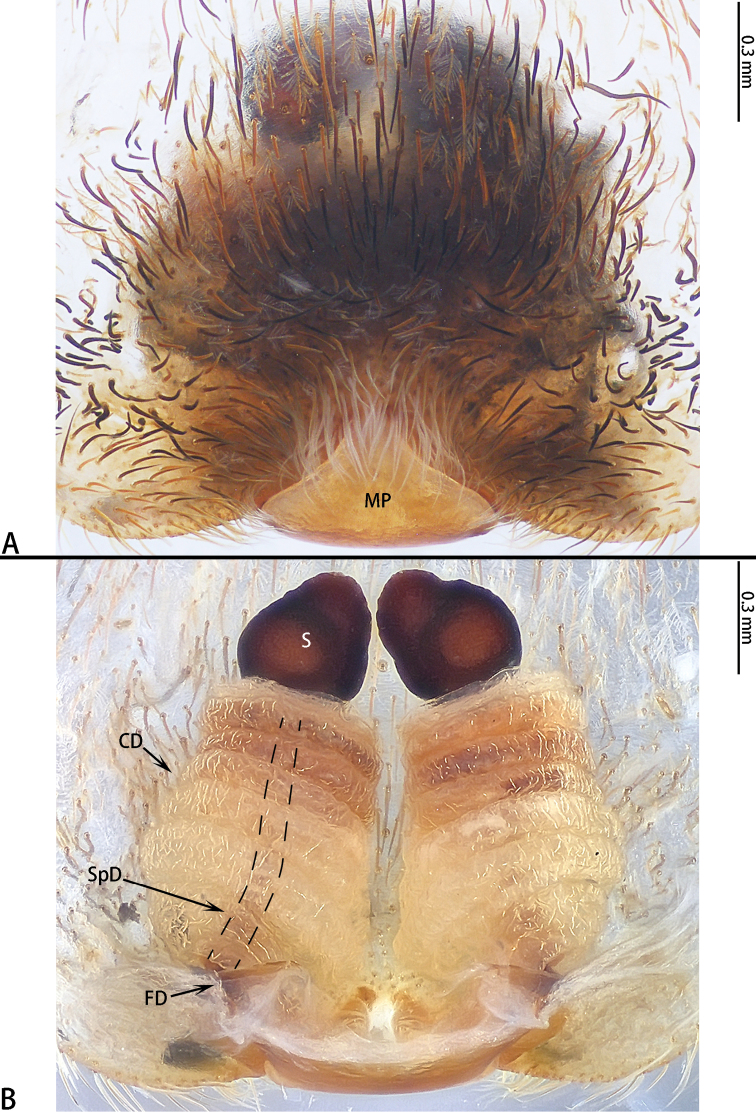
*Asianopis wangi* sp. nov., female paratype. **A** Epigyne **B** Vulva, dorsal view.

Figs 10 , 11 , 12 , 20B , 21C , 22C, D, H , 23

###### Type.

***Holotype*.** ♂ (IZCAS-Ar39681), China, Hainan Province, Wuzhishan City, Wuzhishan Nature Reserve, Diewupo, 17.V.2019, Dongdong Wang leg.

***Paratypes*.** 1♂1♀ (IZCAS-Ar39682-Ar39683), same data as holotype; 1♂2♀ (IZCAS-Ar39684-Ar39686) China, Hainan Province, Wuzhishan City, Nansheng Town, Maoxiang Village, 18.V.2019, Dongdong Wang leg.

###### Etymology.

The species is named after Mr Dongdong Wang, the collector of the holotype; noun (name) in genitive case.

###### Diagnosis.

The males resemble *A. zhuanghaoyuni* sp. nov. but can be distinguished from other species by the ratio of the length of the embolic opening to the length of the embolic tip fold, which is 1:6 in *A. wangi* sp. nov. and 1:8 in *A. zhuanghaoyuni* sp. nov. The fold is more developed in *A. wangi* sp. nov. (Fig. 21C, D). The median plate is triangular in *A. wangi* sp. nov. and subtriangular in *A. zhuanghaoyuni* sp. nov. (Figs 12, 19).

###### Description.

**Male** holotype (Figs 10A, C, D, 11, 20B, 21D, 22C). Total length 15.31, carapace 6.22 long, 4.60 wide, opisthosoma 9.32 long, 2.10 wide. Eye sizes and interdistances: AME 0.30, ALE 0.38, PME 0.65, PLE 0.34, AME–AME 0.30, AME–ALE 0.97, PME–PME 0.23, PME–PLE 0.69, AME–PME 0.24, ALE–PLE 1.82. Clypeus height 0.10. Chelicerae with four promarginal and 10–13 retromarginal teeth. Leg measurements: leg I: 84.08 (21.13 + 26.50 + 29.53 + 6.92), leg II: 59.70 (18.39 + 19.55 + 15.80 + 5.96), leg III: 36.14 (12.05 + 11.79 + 10.26 + 2.04), leg IV: 37.23 (11.92 + 12.37 + 11.28 + 1.66). Leg formula: 1243.

Male palp (Figs 11, 20B, 21D). Cymbium hemispherical; tegulum flat, obscured by embolic coils; embolus long and strongly coiled, originating at 10 o’clock and coiling 1500° around MA; embolic tip widened subapically, strongly folded and without apophysis. MA large, with two lobes.

**Female** paratype (Figs 10B, E, F, 12, 22D). Total length 24.04, carapace 7.56 long, 5.32 wide, opisthosoma 16.28 long, 6.86 wide. Eye sizes and interdistances: AME 0.28, ALE 0.38, PME 1.34, PLE 0.42, AME–AME 0.13, AME–ALE 1.03, PME–PME 0.39, PME–PLE 1.30, AME–PME 0.22, ALE–PLE 1.92. Clypeus height 0.34 (*n* = 1). Chelicerae with four promarginal and 8–13 retromarginal teeth (8(*n* = 1), 13(*n* = 1)). Leg measurements: Leg I: 54.24 (16.22 + 16.83 + 17.63 + 3.56), leg II: 50.59 (15.90 + 16.41 + 15.00 + 3.28), leg III: 30.84 (10.96 + 10.38 + 7.88 + 1.62), leg IV: 30.28 (10.13 + 10.58 + 8.27 + 1.30). Leg formula: 1234.

Epigyne (Fig. 12) with a median plate, CD with 7 or 8 turns, S oval, SpD consistently narrow.

###### Distribution.

China (Hainan).

##### Asianopis wuchaoi

Taxon classificationAnimaliaAraneaeDeinopidae

Lin & Li sp. nov.

4E6A07E0-5DE1-56A3-BD97-D1F0919F2CA0

http://zoobank.org/F05E46B7-98E7-4DA1-B7DF-AD440C2E05B6

**Figure 13.**
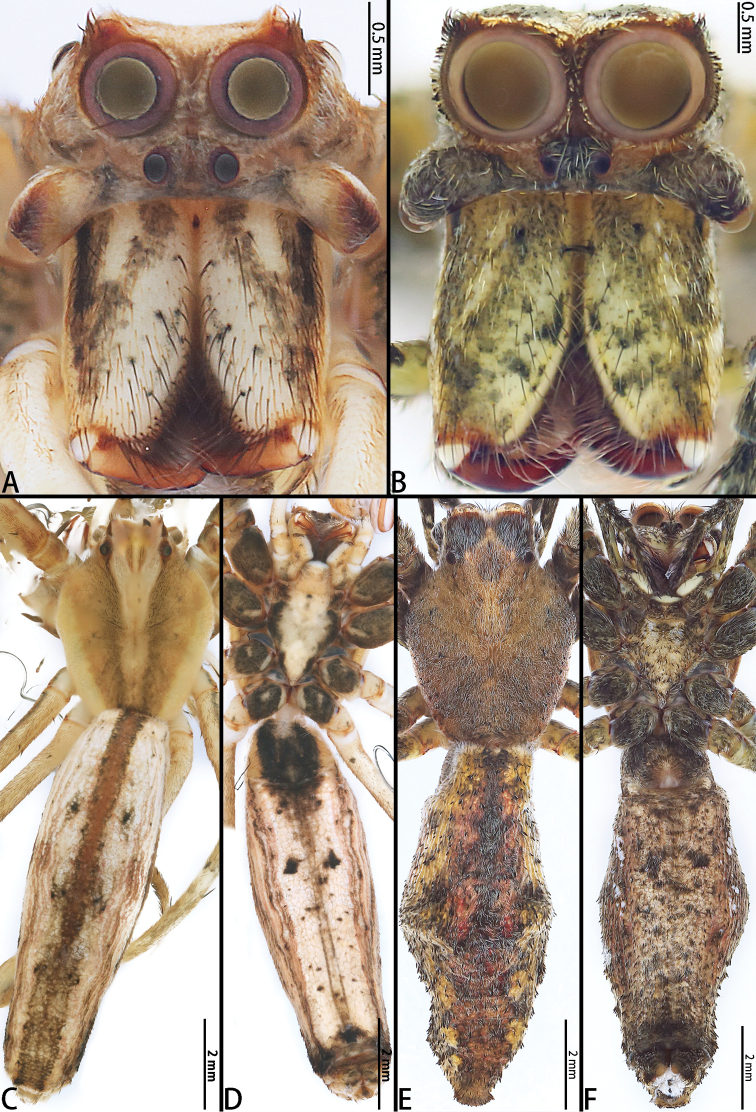
*Asianopis wuchaoi* sp. nov., male holotype and female paratype. **A** Male prosoma, frontal view **B** Female prosoma, frontal view **C** Male habitus, dorsal view **D** Male habitus, ventral view **E** Female habitus, dorsal view **F** Female habitus, ventral view.

**Figure 14.**
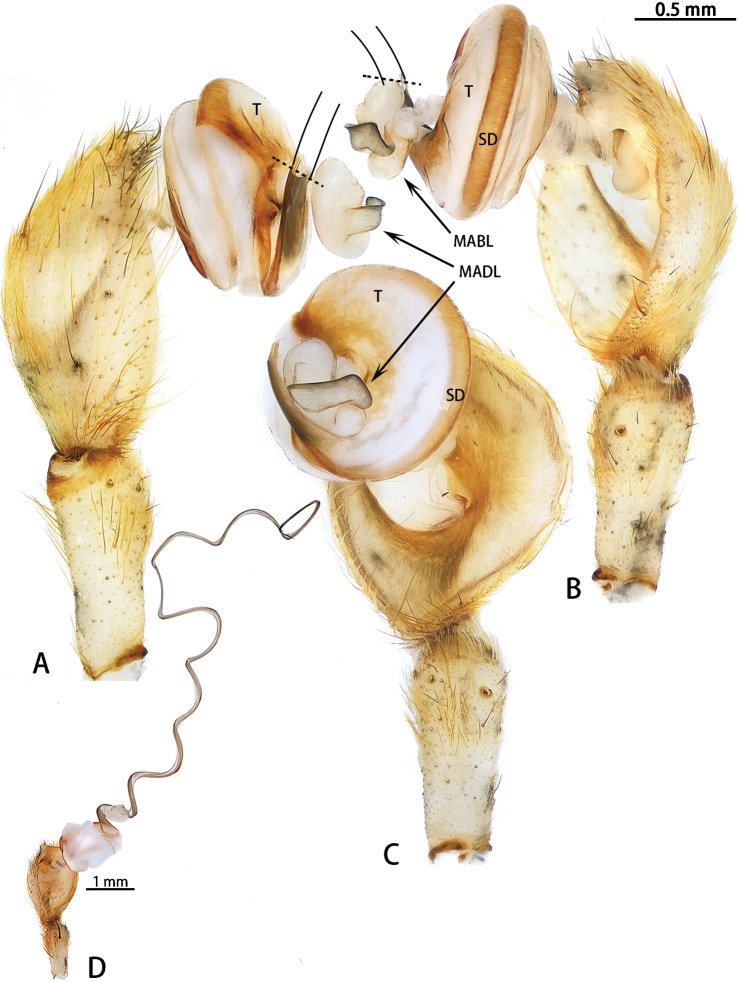
*Asianopis wuchaoi* sp. nov., male holotype. **A** Right palp (flipped horizontally), prolateral view **B** Right palp (flipped horizontally), retrolateral view **C** Left palp, prolateral view **D** Left palp, prolateral view.

**Figure 15.**
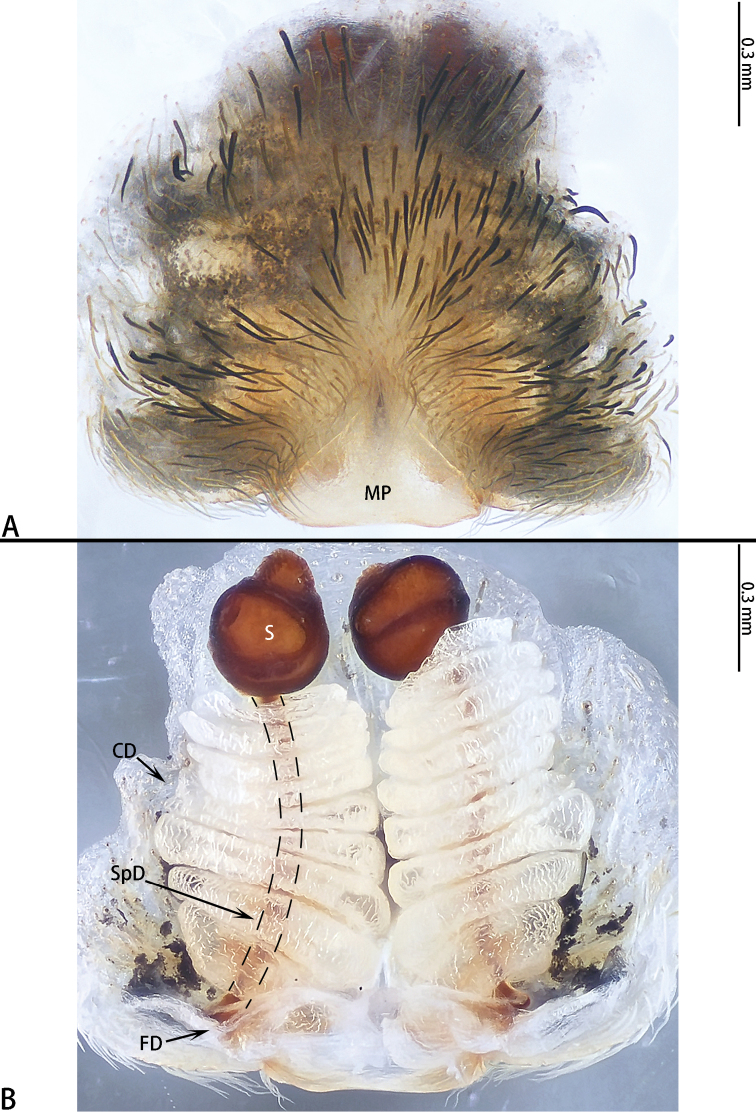
*Asianopis wuchaoi* sp. nov., female paratype. **A** Epigyne **B** Vulva, dorsal view.

Figs 13 , 14 , 15 , 21B , 22B , 23

###### Type.

***Holotype*.** ♂ (IZCAS-Ar39687), China, Yunnan Province, Jinghong City, Mount Jinuo, 10.V.2019, Chao Wu leg.

***Paratypes*.** 2♀ (IZCAS-Ar39688-Ar39689), China, Yunnan Province, Jinghong City, Mengla County, Mengxing Village, 16.VI.2019, Yi Li leg.; 1♀ (IZCAS-Ar39690), China, Yunnan Province, Jinghong City, Situlaozhai Village, 20.V.2019, Chaotai Wei leg.

###### Etymology.

The species is named after Mr Chao Wu, the collector of the holotype male; noun (name) in genitive case.

###### Diagnosis.

The males can be easily distinguished by the length of the palpal tibia which is almost equal to the length of the cymbium; simple embolic tip with ETA (Fig. 21B); embolus coiling almost 3300° around MA. Epigyne with a well-developed, subtriangular median plate, obscureing CO, and CD with 9 turns (Fig. 14).

###### Description.

**Male** holotype (Figs 13A, C, D, 14, 21A). Total length 12.14, carapace 4.00 long, 3.40 wide, opisthosoma 8.14 long, 2.4 wide. Eye sizes and interdistances: AME 0.15, ALE 0.26, PME 0.52, PLE 0.29, AME–AME 0.17, AME–ALE 0.70, PME–PME 0.16, PME–PLE 0.61, AME–PME 0.11, ALE–PLE 0.95. Clypeus height 0.05. Chelicerae with four promarginal and six retromarginal teeth. Leg measurements: leg I: damaged, leg II: damaged, leg III: (6.92 + 6.86 + ? + 1.44), leg IV: 21.82 (6.91 + 7.18 + 6.35 + 1.38).

Male palp (Figs 14, 21A). Cymbium hemispherical; tegulum flat, obscured by embolic coils; embolus long and strongly coiled, originating at five o’clock and coiling 3300° around MA. MA large, with two lobes.

**Female** paratype (Figs 13B, E, F, 15). Total length 14.60, carapace 6.28 long, 4.10 wide, opisthosoma 9.29 long, 3.72 wide. Eye sizes and interdistances: AME 0.11, ALE 0.34, PME 0.94, PLE 0.29, AME–AME 0.30, AME–ALE 1.03, PME–PME 0.06, PME–PLE 0.64, AME–PME 0.14, ALE–PLE 1.33. Clypeus height 0.13 (*n* = 1). Chelicerae with four promarginal and 8–13 retromarginal teeth (8(*n* = 1), 10(*n* = 1), 13(*n* = 1)). Leg measurements: Leg I: 39.82 (12.11 + 11.67 + 13.01 + 3.03), leg II: 36.81 (11.47 + 11.79 + 10.83 + 2.72), leg III: 23.53 (9.47 + 6.79 + 5.83 + 1.44), leg IV: 21.71 (7.18 + 7.76 + 5.70 + 1.07). Leg formula: 1234.

Epigyne (Fig. 15) with a median plate, obscuring CO, CD with 9 turns, S oval, SpD is consistently thin.

###### Distribution.

China (Yunnan).

###### Note.

The male died during ecdysis so some legs are damaged or curled, and the palps are expanded.

##### Asianopis zhuanghaoyuni

Taxon classificationAnimaliaAraneaeDeinopidae

Lin & Li sp. nov.

5184F46B-9ADD-58D6-AAAD-2A7212722DC1

http://zoobank.org/21A5E514-F8EE-4479-9338-51D419AA6E4A

Figs 2D, G, H, J , 16 , 17 , 18 , 20 , 21D , 22E, F, H , 23

###### Type.

***Holotype*.** ♂ (IZCAS-Ar39691), China, Fujian Province, Fuzhou City, Minhou County, Xiyuan Reservoir, 26°03'15.5"N, 119°06'05.4"E, elevation ca 102 m, 25.VI.2018, Haoyun Zhuang and Zhuoheng Jiang leg.

***Paratypes*.** 1♀ (IZCAS-Ar39692), same data as holotype, Haoyun Zhuang leg.; 1♂1♀ (IZCAS-Ar39693-Ar39694), same locality data as holotype, but 15.V.2018, Haoyun Zhuang leg.; 1♂4♀ (IZCAS-Ar39695-Ar39699), same locality data as holotype, but 19.VI.2019, Haoyun Zhuang leg.; 1♂1♀ (IZCAS-Ar39700-Ar39701), same locality data as holotype, but 26.V.2019, Haoyun Zhuang leg.

###### Etymology.

The species is named after Mr Haoyun Zhuang, the collector of the type specimens; noun (name) in genitive case.

###### Diagnosis.

The males resemble *A. konplong* (Logunov, 2018) comb. nov. but can be distinguished by the embolus originating at five o’clock in *A. zhuanghaoyuni* sp. nov. (9 o’clock in *A. konplong* (Logunov, 2018) comb. nov.); the ratio of the length of the palpal tarsus to the length of the cymbium is 11:9 in *A. zhuanghaoyuni* sp. nov., while in *A. konplong* (Logunov, 2018), comb. nov. it is 1:1 (Figs 18, 22A; Logunov 2018, figs 4–6).

###### Description.

**Figure 16.**
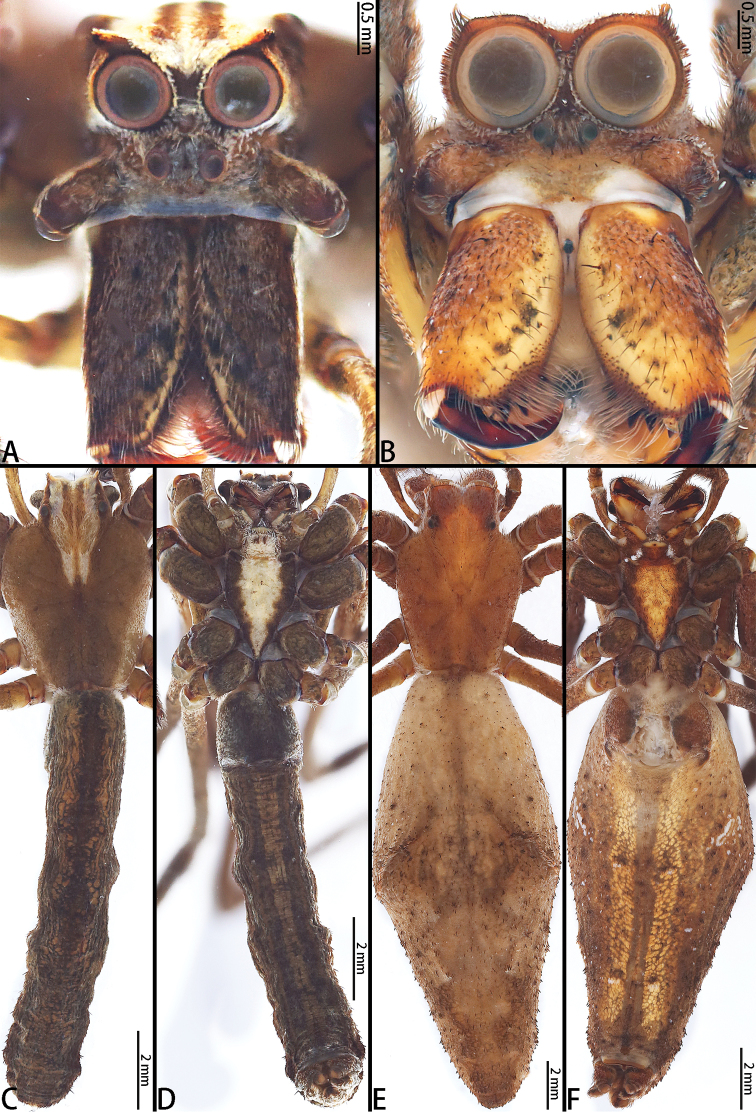
*Asianopis zhuanghaoyuni* sp. nov., male holotype and female paratype. **A** Male prosoma, frontal view **B** Female prosoma, frontal view **C** Male habitus, dorsal view **D** Male habitus, ventral view **E** Female habitus, dorsal view **F** Female habitus, ventral view.

**Figure 17.**
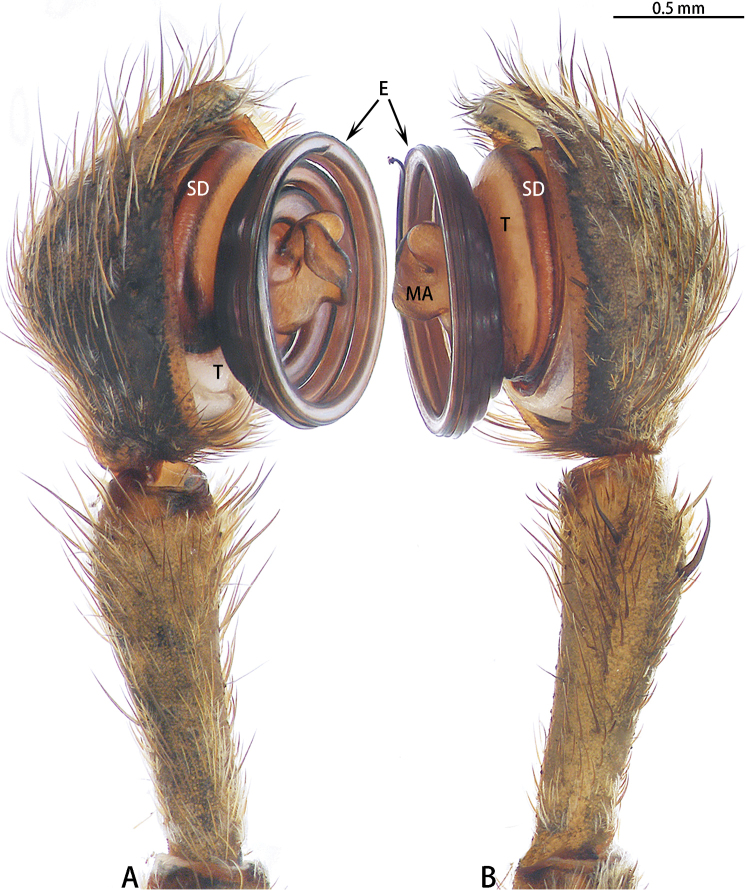
*Asianopis zhuanghaoyuni* sp. nov., male holotype, left palp. **A** Prolateral view **B** Retrolateral view.

**Male** holotype (Figs 2G, 16A, C, D, 17, 20A, 21E, 22E). Total length 16.54, carapace 5.58 long, 3.84 wide, opisthosoma 11.40 long, 1.90 wide. Eye sizes and interdistances: AME 0.25, ALE 0.30, PME 0.59, PLE 0.30, AME–AME 0.25, AME–ALE 0.85, PME–PME 0.23, PME–PLE 0.59, AME–PME 0.19, ALE–PLE 1.28. Clypeus height 0.20. Chelicerae with four promarginal teeth and a retromarginal tooth. Leg measurements: leg I: 66.35 (18.50 + 22.55 + 18.95 + 6.35), leg II: 52.87 (16.54 + 17.65 + 13.10 + 5.58), leg III: 30.39 (10.78 + 10.83 + 7.18 + 1.60), leg IV: 30.06 (10.42 + 11.12 + 7.18 + 1.34). Leg formula: 1234.

**Figure 18.**
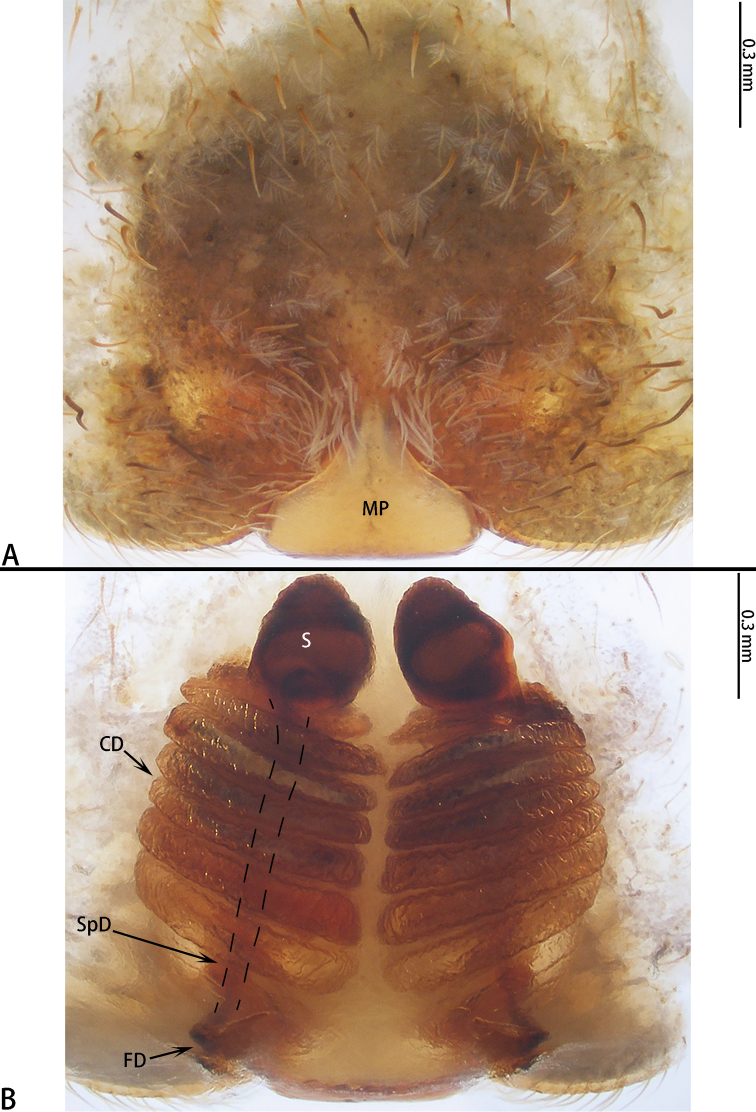
*Asianopis zhuanghaoyuni* sp. nov., female paratype. **A** Epigyne **B** Vulva, dorsal view.

Male palp (Figs 18, 22A). Cymbium hemispherical; tegulum flat, obscured by embolus coils; originating at five o’clock, coiling 1500° around MA, embolic tip widened subapically, folded and without apophysis. MA large, with two lobes.

**Figure 19.**
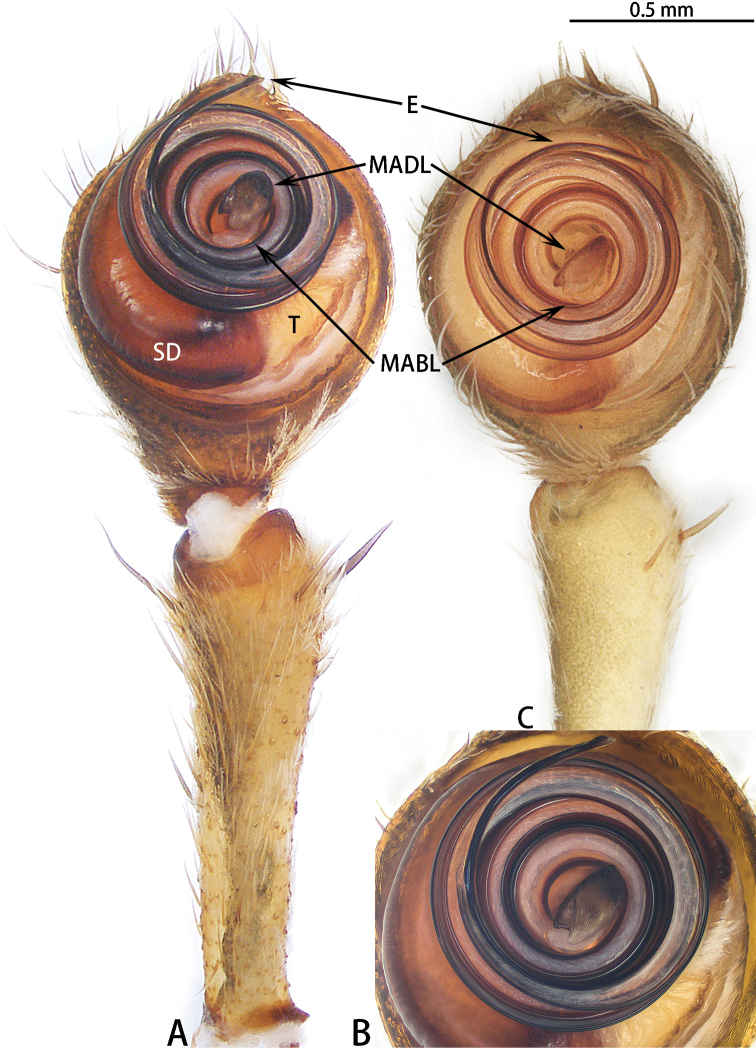
*Asianopis liukuensis* comb. nov., left palp, ventral view. **A, B** Male from Xishuangbanna **C** Male from India, type of *Deinopis scrubjunglei* syn. nov.

**Figure 20.**
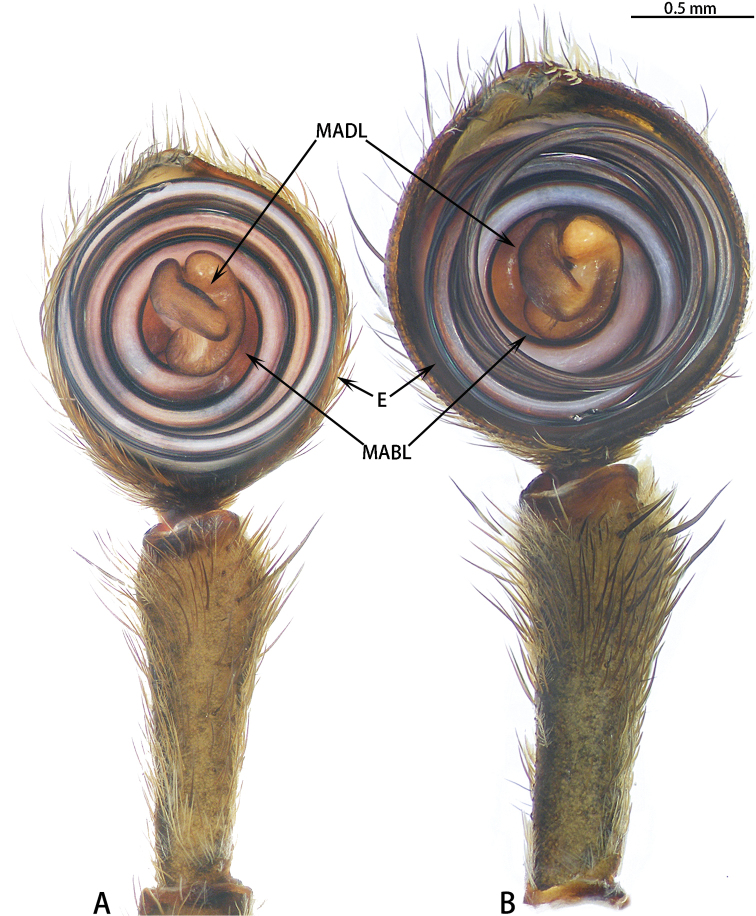
Ventral view of left palp, holotype males. **A***A. zhuanghaoyuni* sp. nov. **B***A. wangi* sp. nov.

**Figure 21.**
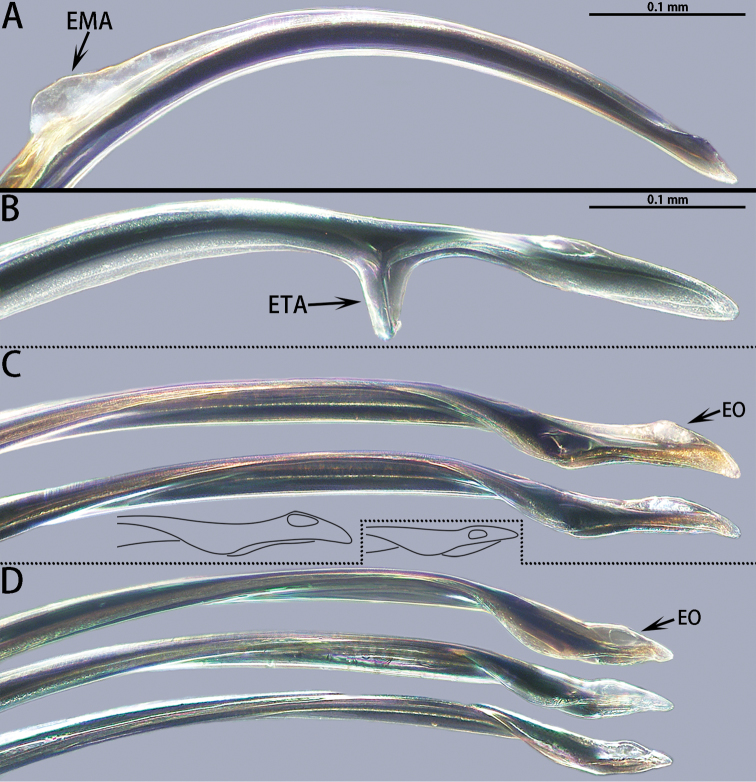
Embolic tips of four species of *Asianopis* gen. nov. **A***A. liukuensis* (Yin, Griswold & Yan, 2002) comb. nov. **B***A. wuchaoi* sp. nov. **C***A. zhuanghaoyuni* sp. nov. **D***A. wangi* sp. nov.

**Figure 22.**
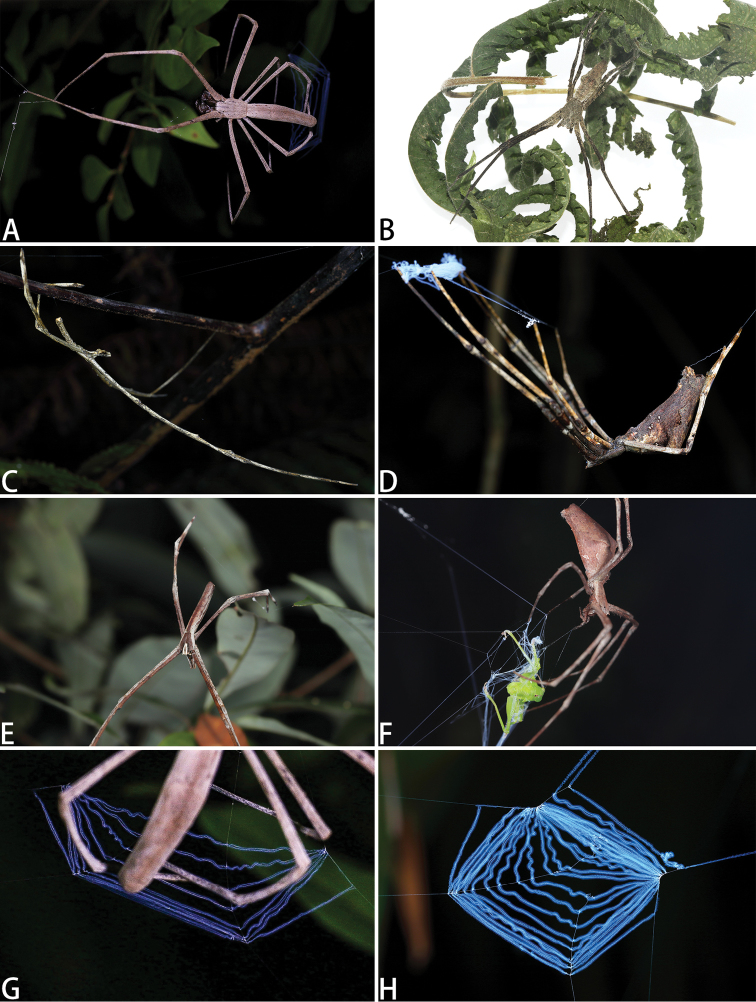
Photos of four live spiders of *Asianopis* gen. nov., including webs of two species of *Asianopis* gen. nov. **A***A. liukuensis* comb. nov., female **B***A. wuchaoi* sp. nov., female **C***A. wangi* sp. nov., male **D***A. wangi* sp. nov., female **E***A. zhuanghaoyuni* sp. nov., male **F***A. zhuanghaoyuni* sp. nov., female **G** Web of *A. liukuensis* comb. nov. **H** Web of *A. wangi* sp. nov.

**Figure 23.**
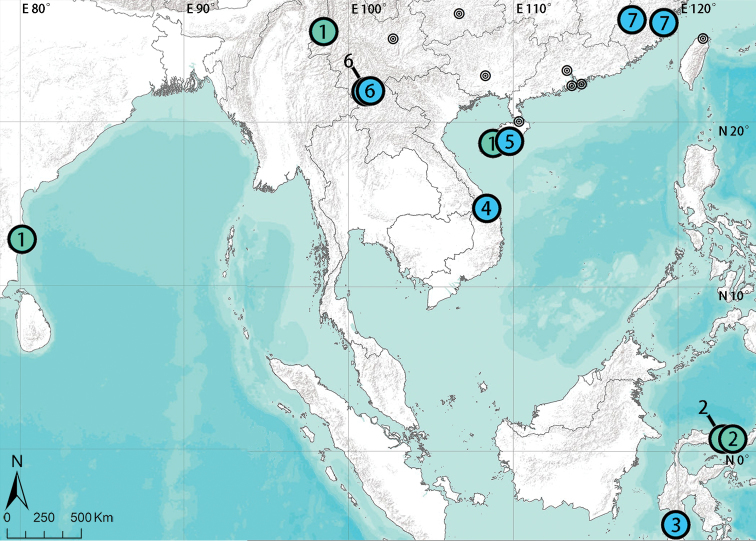
Distribution records of seven species of *Asianopis* gen. nov. in Asia. **1***A. liukuensis* comb. nov. **2***A. dumogae* sp. reval. comb. nov. **3***A. celebensis* comb. nov. **4***A. konplong* comb. nov. **5***A. wangi* sp. nov. **6***A. wuchaoi* sp. nov. **7***A. zhuanghaoyuni* sp. nov.

**Female** paratype (Figs 2H, J, 16B, E, F, 18, 22F, H). Total length 22.60, carapace 5.90 long, 4.55 wide, opisthosoma 15.40 long, 5.90 wide. Eye sizes and interdistances: AME 0.22, ALE 0.35, PME 1.08, PLE 0.33, AME–AME 0.37, AME–ALE 1.22, PME–PME 0.16, PME–PLE 0.98, AME–PME 0.081, ALE–PLE 1.61. Clypeus height 0.59. (*n* = 1). Chelicerae with four promarginal and 10 or 11 (10 (*n* = 2), 11 (*n* = 1)) retromarginal teeth. Leg measurements: Leg I: 49.68 (14.80 + 15.83 + 16.02 + 3.03), leg II: 46.08 (14.71 + 15.20 + 13.33 + 2.84), leg III: 27.79 (9.73 + 9.41 + 7.18 + 1.47), leg IV: 26.78 (9.02 + 9.61 + 6.86 + 1.29). Leg formula: 1234.

Epigyne (Fig. 18) with a median plate, CD with 7–8 turns, S oval, SpD consistently thin.

###### Distribution.

China (Fujian).

## Supplementary Material

XML Treatment for Asianopis

XML Treatment for Asianopis dumogae

XML Treatment for Asianopis liukuensis

XML Treatment for Asianopis celebensis

XML Treatment for Asianopis konplong

XML Treatment for Asianopis wangi

XML Treatment for Asianopis wuchaoi

XML Treatment for Asianopis zhuanghaoyuni

## Supplementary materials

Supplementary material 1 *Asianopis* gen. nov., a new genus of the spider family Deinopidae from Asia Data type: specimen/primer/DNA sequence Explanation note: **Table S1.** List of voucher information and GenBank accession numbers. **Table S2.** Primers and PCR conditions for the genetic markers used in this study (modified after Zhao and Li unpublished). **Table S3.** Sequence characteristics and models of DNA evolution selected for the seven sequence regions analyzed.References cited in supplementary tables. https://binary.pensoft.net/file/380036This dataset is made available under the Open Database License (http://opendatacommons.org/licenses/odbl/1.0/). The Open Database License (ODbL) is a license agreement intended to allow users to freely share, modify, and use this Dataset while maintaining this same freedom for others, provided that the original source and author(s) are credited.Yejie Lin, Lili Shao, Ambros Hänggi, John T.D. Caleb, Joseph K.H. Koh, Peter Jäger, Shuqiang Li

## References

[B1] Caleb JTD Mathai MT (2014) A new species of *Deinopis* MacLeay (Araneae: Deinopidae) from India. Indian Journal of Arachnology 3(1): 1–7.

[B2] Capella-Gutiérrez S Silla-Martínez JM Gabaldón T (2009) TrimAl: a tool for automated alignment trimming in large-scale phylogenetic analyses. Bioinformatics 25: 1972–1973. 10.1093/bioinformatics/btp348 19505945 PMC2712344

[B3] Chamberland L McHugh A Kechejian S Binford GJ Bond JE Coddington JA Dolman G Hamilton CA Harvey MS Kuntner M Agnarsson I (2018) From Gondwana to GAARlandia: evolutionary history and biogeography of ogre-faced spiders (*Deinopis*). Journal of Biogeography 45: 2442–2457. 10.1111/jbi.13431

[B4] Coddington JA Kuntner M Opell BD (2012) Systematics of the spider family Deinopidae with a revision of the genus *Menneus*. Smithsonian Contributions to Zoology 636: 1–61. 10.5479/si.00810282.636.1

[B5] Doleschall L (1859) Tweede Bijdrage tot de kennis der Arachniden van den Indischen Archipel. Acta Societatis Scientiarum Indica-Neerlandica 5: 1–60.

[B6] Griswold CE Ramírez MJ Coddington JA Platnick NI (2005) Atlas of phylogenetic data for entelegyne spiders (Araneae: Araneomorphae: Entelegynae) with comments on their phylogeny. Proceedings of the California Academy of Sciences 56 (Supplement II): 1–324.

[B7] Katoh K Standley DM (2013) MAFFT. Multiple sequence alignment software version 7: improvements in performance and usability. Molecular Phylogenetics and Evolution 30: 772–780. 10.1093/molbev/mst010 PMC3603318 23329690

[B8] Kumar S Stecher G Tamura K (2016) MEGA7: Molecular Evolutionary Genetics Analysis version 7.0 for bigger datasets. Molecular Biology and Evolution 33(7): 1870–1874. 10.1093/molbev/msw054 27004904 PMC8210823

[B9] Lanfear R Calcott B Ho SY Guindon S (2012) Partitionfinder: combined selection of partitioning schemes and substitution models for phylogenetic analyses. Molecular Biology and Evolution 29: 1695–1701. 10.1093/molbev/mss020 22319168

[B10] Logunov DV (2018) A new ogre-faced spider species of the genus *Deinopis* MacLeay, 1839 from Vietnam (Aranei: Deinopidae). Arthropoda Selecta 27(2): 139–142. 10.15298/arthsel.27.2.05

[B11] MacLeay WS (1839) On some new forms of Arachnida Annals of Natural History (Series 1) 2(7): 1–14. [pls 1, 2] 10.1080/00222933809496646

[B12] Merian P (1911) Die Spinnenfauna von Celebes. Beiträge zur Tiergeographie im Indoaustralischen Archipel. Zoologische Jahrbücher, Abteilung für Systematik, Geographie und Biologie der Tiere 31: 165–354.

[B13] Rambaut A Suchard MA Xie D Drummond AJ (2014) Tracer v1.6. http://tree.bio.ed.ac.uk/software/tracer [Accessed on: 2019–12–31]

[B14] Roewer CF (1938) Araneae. Résultats scientifiques du Voyage aux indes orientales néerlandaises de la SS. AA. RR. le Prince et la Princesse Leopold de Belgique. Mémoires du Musée Royal d’Histoire Naturelle de Belgique 3(19): 1–94.

[B15] Ronquist F Teslenko M van der Mark P Ayres DL Darling A Hohna S Larget B Liu L Suchard MA Huelsenbeck JP (2012) MrBayes 3.2: efficient Bayesian phylogenetic inference and model choice across a large model space. Systematic Biology 61: 539–542. 10.1093/sysbio/sys029 22357727 PMC3329765

[B16] Simon E (1876) Etude sur le arachnides du Congo. Bulletin de la Société Zoologique de France 1: 12–15, 215–224.

[B17] Simon E (1909) Etude sur les arachnides du Tonkin (1re partie). Bulletin Scientifique de la France et de la Belgique 42: 69–147. 10.5962/bhl.part.24151

[B18] Stamatakis A (2014) RAxML version 8: a tool for phylogenetic analysis and post-analysis of large phylogenies. Bioinformatics 30: 1312–1313. 10.1093/bioinformatics/btu033 24451623 PMC3998144

[B19] World Spider Catalog (2019) World Spider Catalog, version 20.5. Natural History Museum, Bern. http://wsc.nmbe.ch [Accessed on: 2019–12–21]

[B20] Yin CM Griswold CE Yan HM (2002) A new ogre-faced spider (*Deinopis*) from the Gaoligong Mountains, Yunnan, China (Araneae, Deinopidae). Journal of Arachnology 30: 610–612. 10.1636/0161-8202(2002)030[0610:ANOFSD]2.0.CO;2

