# Supplementary material for: Asianopis gen. nov., a new genus of the spider family Deinopidae from Asia
Source: Zookeys. 2020 Feb 12;911:67–99. doi: 10.3897/zookeys.911.38761 (PMC7031397; doi:10.3897/zookeys.911.38761)
Supplement: Supplementary material 1 [file zookeys-911-067-s001.doc]

**Supplementary tables:**

**Table S1.** List of voucher information and GenBank accession numbers.

| Taxon | Voucher | Location | GenBank Accession Number | | | | | | |
| --- | --- | --- | --- | --- | --- | --- | --- | --- | --- |
|  |  |  | 28S | 18S | 12S | 16S | COI | H3 | wnt |
| *Uloborus glomosus* | NA | NA | EU003437,EU003438 | NEU003366 | NA | NA | EU003310 | EU003340 | NA |
| *Deinopis sp.FAPDNA006* | NA | Dominica | EU003403,EU003403 | EU003382, EU003383 | NA | EU003249 | NA | NA | NA |
| *Deinopis spinosa* | NA | America | FJ525370 | FJ525388 | NA | FJ525351 | FJ525318 | FJ525337 | FJ607634 |
| *Deinopis sp. SP68* | NA | South America | KY017019 | KY016395 | NA | KY015817 | KY017667 | KY018180 | NA |
| *Deinopis sp. GH4* | NA | Central America | KY017018 | KY016394 | KY015359 | KY015816 | KY017666 | NA | NA |
| *Menneus camelus* | NA | South Africa | KC848997 | KC848946 | KC848916 | KC849122 | KC849079 | KC849038 | NA |
| *Menneus capensis* | NA | NA | KY017022 | NA | KY015362 | KY015819 | KY017670 | NA | NA |
| *Menneus sp. LB-2014a* | NA | NA | KM486379 | KM486167 | KM486222 | KM486311 | KM486453 | KM486498 | NA |
| Deinopidae sp. Africa | SL25 | Madagascar | MN332014 | MN331862 | MN331949 | MN331887 | NA | MN257998 | MN258020 |
| *Asianopis liukuensis* | SL26 | Vietnam | MN332015 | MN331863 | MN331950 | MN331888 | MN257976 | MN257999 | MN258021 |
| *Asianopis liukuensis* | ly38 | Yunnan, China | MN332006 | NA | NA | MN331879 | MN257968 | MN257991 | MN258012 |
| *Asianopis liukuensis* | ly43 | Guangxi, China | MN332011 | NA | NA | MN331884 | MN257973 | NA | MN258017 |
| *Asianopis wuchaoi* | ly36 | Yunnan, China | MN332005 | MN331855 | MN331943 | MN331877 | MN257966 | MN257989 | MN258010 |
| *Asianopis wuchaoi* | ly37 | Yunnan, China | NA | MN331856 | MN331944 | MN331878 | MN257967 | MN257990 | MN258011 |
| *Asianopis wuchaoi* | YNZ | Yunnan, China | NA | MN331865 | MN331952 | NA | MN257978 | MN258001 | NA |
| *Asianopis wuchaoi* | YNM | Yunnan, China | NA | MN331864 | MN331951 | NA | MN257977 | MN258000 | NA |
| *Asianopis wangi* | ly33 | Hainan, China | MN332002 | MN331852 | MN331940 | MN331874 | MN257963 | MN257986 | MN258007 |
| *Asianopis wangi* | ly39 | Hainan, China | MN332007 | MN331857 | MN331945 | MN331880 | MN257969 | MN257992 | MN258013 |
| *Asianopis wangi* | ly41 | Hainan, China | MN332009 | MN331859 | MN331947 | MN331882 | MN257971 | MN257994 | MN258015 |
| *Asianopis wangi* | ly42 | Hainan, China | MN332010 | MN331860 | MN331948 | MN331883 | MN257972 | MN257995 | MN258016 |
| *Asianopis wangi* | ly44 | Hainan, China | MN332012 | MN331861 | NA | MN331885 | MN257974 | MN257996 | MN258018 |
| *Asianopis wangi* | ly45 | Hainan, China | MN332013 | NA | NA | MN331886 | MN257975 | MN257997 | MN258019 |
| *Asianopis zhuanghaoyuni* | ly34 | Fujian, China | MN332003 | MN331853 | MN331941 | MN331875 | MN257964 | MN257987 | MN258008 |
| *Asianopis zhuanghaoyuni* | ly35 | Hong Kong, China | MN332004 | MN331854 | MN331942 | MN331876 | MN257965 | MN257988 | MN258009 |
| *Asianopis zhuanghaoyuni* | ly40 | Fujian, China | MN332008 | MN331858 | MN331946 | MN331881 | MN257970 | MN257993 | MN258014 |
| *Asianopis zhuanghaoyuni* | FJ11 | Fujian, China | MN331995 | MN331845 | MN331933 | MN331867 | MN257956 | MN257979 | NA |
| *Asianopis zhuanghaoyuni* | FJ12 | Fujian, China | MN331996 | MN331846 | MN331934 | MN331868 | MN257957 | MN257980 | NA |
| *Asianopis zhuanghaoyuni* | FJ13 | Fujian, China | MN331997 | MN331847 | MN331935 | MN331869 | MN257958 | MN257981 | MN258002 |
| *Asianopis zhuanghaoyuni* | FJ14 | Fujian, China | MN331998 | MN331848 | MN331936 | MN331870 | MN257959 | MN257982 | MN258003 |
| *Asianopis zhuanghaoyuni* | FJ21 | Fujian, China | MN331999 | MN331849 | MN331937 | MN331871 | MN257960 | MN257983 | MN258004 |
| *Asianopis zhuanghaoyuni* | FJ22 | Fujian, China | MN332000 | MN331850 | MN331938 | MN331872 | MN257961 | MN257984 | MN258005 |
| *Asianopis zhuanghaoyuni* | FJ31 | Fujian, China | MN332001 | MN331851 | MN331939 | MN331873 | MN257962 | MN257985 | MN258006 |

**Table S2.** Primers and PCR conditions for the genetic markers used in this study (modified after Zhao and Li unpublished).

| Gene |  | Sequence | PCR conditions  (annealing temperature and cycle number) | Reference |
| --- | --- | --- | --- | --- |
| 12S rRNA | 12SaF  12SbF  12SR | 5′–GACAAAATTCGTGCCAGC–3′  5′–AAACTAGGATTAGATACCCTATTAT–3′  5′–AAGAGCGACGGGCGATGTGT–3′ | 45 °C (5) + 52 °C (30)  45 °C (5) + 52 °C (30) | Simon et al., 1994 |
| 16S rRNA | 16SF  16SR | 5′–CGCCTGTTTATCAAAAACAT–3′  5′–CTCCGGTTTGAACTCAGATCA–3′ | 45 °C (5) + 48 °C (30)/ 45 °C (35) | Tan et al., 1999 |
| 18S rRNA | 18S1F  18S1R  18S3F  18S3R  18S2F  18S2R | 5′–TACCTGGTTGATCCTGCCAGTAG–3′  5′–GTGGTGCCCTTCCGTCAATT–3′  5′–GCGAAAGCATTTGCCAAGAA–3′  5′–GATCCTTCCGCAGGTTCACCTAC–3′  5′–GTTCGATTCCGGAGAGGGA–3′  5′–GCATCACAGACCTGTTATTGC–3′ | 45 °C (35)  45 °C (35)  45 °C (35) | Giribet et al., 1996 |
| 28S rRNA | 28S1F  28S1R  28S2aF  28S2aR  28S2bF  28S2bR  28S3F  28S3R | 5′–ACCCGCTGAATTTAAGCATAT–3′  5′–ATCTGACGATCGATTTGCAC–3′  5′–GACCCGTCTTGAAACACGGA–3′  5′–GCTACTACCACCAAGATCTGCA–3′  5′–GCGAGTAGATCGGTCACCCA–3′  5′–AGTTGTTACACACTCCTTGGC–3′  5′–ACCTATTCTCAAACTTTAAATGG–3′  5′–GACTTCCCTTACCTACAT–3′ | 48 °C (35)  48 °C (35)  48 °C (35)  45 °C (35) | Bond et al., 2006  Maddison et al., 2007  Whiting et al., 1997  Schnare et al., 1996  Unpublished work, designed by Huifeng Zhao  Whiting et al., 1997 |
| COI | COI1490  COI2198 | 5′–GGTCAACAAATCATAAAGATATTGG–3′  5′–TAAACTTCAGGGTGACCAAAAAATCA–3′  5′–GGAGGATTTGGAAATTGATTAGTTCC–3′  5′–GGATAATCAGAATATCGTCGAGG–3′ | 45 °C (35)  45 °C (35) | Folmer et al., 1994  Simon et al., 1994  Hedin et al., 2001 |
| H3 | H3aF  H3aR | 5′–ATGGCTCGTACCAAGCAGACVGC–3′  5′–ATATCCTTRGGCATRATRGTGAC–3′ | 45 °C (5) + 48 °C (30)/ 45 °C (35) | Colgan et al., 1998 |
| wnt (first run usingWnt1F  and Wnt1R, the PCR product was used as the template for the second run with Wnt2F  and Wnt2R) | Wnt1F  Wnt1R  Wnt2F  Wnt2R | 5′–GYAAATGCCAYGGWATGTCMGG–3′  5′–ACTTGRCAACACCARTGAAAWG–3′  5′–CNGTTCAAACTTGYTGGATG–3′  5′–CAGTGRAATGTRCARTTGC–3′ | 45 °C (35)  46 °C (35)/ 48 °C (35) | Blackledge et al., 2009  Unpublished work, designed by Fengyuan Li |

**Table S3.** Sequence characteristics and models of DNA evolution selected for the seven sequence regions analyzed

| Genes | Aligned length (bp) | | Best-fit model selected by PartitionFinder | Subset Partitions |
| --- | --- | --- | --- | --- |
| 12S | 316 | GTR+G | | 12S, 16S |
| 16S | 433 | GTR+G | |
| 18S | 1584 | GTR+I+G | | 18S |
| 28S | 1228 | GTR+I+G | | 28S |
| COI | 639 | GTR+G | | COI |
| H3 | 360 | SYM+G | | H3 |
| wnt | 333 | K80+G | | wnt |

**References cited in supplementary tables**

Blackledge TA, Scharff N, Coddington JA, Szüts T, Wenze JW, Hayashi CY, Agnarsson I (2009) Reconstructing web evolution and spider diversification in the molecular era. Proceedings of the National Academy of Sciences of the United States of America 106 (13): 5229–5234. <https://doi.org/10.1073/pnas.0901377106>

Bond JE, Hedin M (2006) A total evidence assessment of the phylogeny of North American euctenizine trapdoor spiders (Araneae, Mygalomorphae, Cyrtaucheniidae) using Bayesian inference. Molecular Phylogenetics and Evolution 4 (1): 70–85. <https://doi.org/10.1016/j.ympev.2006.04.026>

Colgan DJ, McLauchlan A, Wilson GDF, Livingston SP, Edgecombe GD, Macaranas J, Cassis G, Gray MR (1998) Histone H3 and U2 snRNA DNA sequences and arthropod molecular evolution. Australian Journal of Zoology 46 (5): 419–437. <https://doi.org/10.1071/zo98048>

Folmer O, Black M, Hoeh W, Lutz R, Vrijenhoek R (1994) DNA primers for amplification of mitochondrial cytochrome c oxidase subunit I from diverse metazoan invertebrates. Molecular Marine Biology and Biotechnology 3 (5): 294–299.

Giribet G, Carranza S, Baguñà J, Riutort M, Ribera C (1996) First molecular evidence for the existence of a Tardigrada + Arthropoda clade. Molecular Phylogenetics and Evolution 13 (1): 76–84. <https://doi.org/10.1093/oxfordjournals.molbev.a025573>

Hedin MC, Madison WP (2001) A combined molecular approach to phylogeny of the jumping spider subfamily Dendryphantinae (Araneae: Salticidae). Molecular Phylogenetics and Evolution 18 (3): 386–403. <https://doi.org/10.1006/mpev.2000.0883>

Maddison WP, Zhang JX, Bodner MR (2007) A basal phylogenetic placement for the salticid spider *Eupoa*, with descriptions of two new species (Araneae: Salticidae). Zootaxa 1432: 23–33. <https://doi.org/10.11646/zootaxa.1432.1.2>

Schnare MN, Damberger SH, Gray MW, Gutell RR (1996) Comprehensive comparison of structural characteristics in eukaryotic cytoplasmic large subunit (23 S-like) ribosomal RNA. Journal of Molecular Biology 256 (4): 701–719. <https://doi.org/10.1006/jmbi.1996.0119>

Simon C, Frati F, Beckenbach A, Crespi B, Liu H, Flook P (1994) Evolution, weighting, and phylogenetic utility of mitochondrial gene sequences and a compilation of conserved polymerase chain reaction primers. Annals of the Entomological Society of America 87 (6): 651–701. <https://doi.org/10.1093/aesa/87.6.651>

Tan AM, Gillespie RG, Oxford GS (1999) Paraphyly of the *Enoplognatha* group (Araneae, Theridiidae) based on DNA sequences. Journal of Arachnology 27 (2): 481–488.

Whiting MF, Carpenter JC, Wheeler QD, Wheeler WC (1997) The Strepsiptera problem: phylogeny of the holometabolous insect orders inferred from 18S and 28S ribosomal DNA sequences and morphology. Systematic Biology 46 (1): 1–68. <https://doi.org/10.1093/sysbio/46.1.1>
